# X-Linked Inhibitor of Apoptosis Protein (XIAP) Contributes to ERK1/2-Mediated Anoikis Resistance in Hepatocellular Carcinoma

**DOI:** 10.1002/mog2.70059

**Published:** 2026-03-25

**Authors:** Qingyu Zeng, Zun Mao, Sumin Sun, Zhixiang Gao, Junpeng Mu, Zhaoji Pan, Qing Li, Xueyuan Mao, Jianbo Xu, Dousheng Bai, Shile Huang, Long Chen

**Affiliations:** 1 Jiangsu Key Laboratory for Molecular and Medical Biotechnology, College of Life Sciences, Nanjing Normal University, Nanjing, China; 2 Institute of Photomedicine, Shanghai Skin Disease Hospital, School of Medicine, Tongji University, Shanghai, China; 3 Department of Hepatobiliary Surgery, Northern Jiangsu People’s Hospital, Yangzhou, China; 4 Research Institute of General Surgery, Jinling Hospital, School of Medicine, Nanjing University, Nanjing, China; 5 Clinical Laboratory, Xuzhou Central Hospital, Southeast University Affiliated Xuzhou Central Hospital, Xuzhou, China; 6 Department of Pathology, Xuzhou Central Hospital, Xuzhou Clinical School of Xuzhou Medical University, Xuzhou, China; 7 Department of Pathology, The Suqian Clinical College of Xuzhou Medical University, Suqian, China; 8 Department of Hepatobiliary Surgery, The Affiliated Huaian No. 1 People’s Hospital of Nanjing Medical University, Huai’an, China; 9 Department of Biochemistry and Molecular Biology, Louisiana State University Health Sciences Center, Shreveport, Louisiana, USA; 10 Department of Hematology and Oncology, Louisiana State University Health Sciences Center, Shreveport, Louisiana, USA; 11 Feist-Weiller Cancer Center, Louisiana State University Health Sciences Center, Shreveport, Louisiana, USA

**Keywords:** anoikis, extracellular signal-regulated kinases 1/2, hepatocellular carcinoma, resistance, X-linked inhibitor of apoptosis protein

## Abstract

X-linked inhibitor of apoptosis protein (XIAP) is a multifunctional protein that regulates many cellular functions. Anoikis resistance is necessary for hepatocellular carcinoma (HCC) intrahepatic spread and extrahepatic metastasis. However, limited information is available regarding the mechanism of XIAP underlying the resistance of HCC cells to anoikis. Here we show an increased expression of XIAP in microvascular tumor thrombosis (MVTT) of HCC, which is positively correlated with the expression of extracellular signal-regulated kinases 1/2 (ERK1/2). HCC cells exhibit an increase in XIAP expression when detached, leading to their resistance to anoikis. Furthermore, enhanced phosphorylation of XIAP promotes resistance to anoikis in HCC cells. In addition, the zinc-binding baculovirus IAP repeat (BIR) domains of XIAP, instead of the highly intriguing novel gene (RING) domain, are responsible for conferring resistance to anoikis in HCC cells. Importantly, our findings indicate that ERK1/2 can control the expression of XIAP, leading to the resistance of HCC to anoikis in cell-based and mouse models. The significance of the interaction between XIAP and ERK1/2 in resisting anoikis is emphasized by our discoveries, presenting novel avenues for HCC treatment.

## 1 | Introduction

Hepatocellular carcinoma (HCC) is one of the most prevalent solid malignancies globally [1]. It is commonly held that metastasis, a complicated procedure involving various stages, constitutes a pivotal stage in cancer progression and continues to be the primary hindrance in enhancing prognosis and longterm survival rates [2]. Most human cells require anchorage for survival, and when there is improper cell adhesion or absence of extracellular matrix (ECM), the cells are unable to sustain the pro-survival signals, resulting in a reduction of anti-apoptotic signals and triggering the process of anoikis [3, 4]. Increasing evidence indicates that anoikis resistance is necessary for intrahepatic spread and extrahepatic metastasis of HCC cells [5–7]. Hence, exploring the molecular mechanisms that cause resistance to anoikis in HCC could formulate improved treatment plans for HCC patients.

The activity of caspases is vital for controlling anoikis [8, 9]. X-linked inhibitor of apoptosis protein (XIAP), which is the most extensively studied member of IAPs, is widely acknowledged as a vital controller of caspases, directly inhibiting cell death [10]. Relevant studies have confirmed that the zinc-binding baculovirus IAP repeat 2 (BIR2) domain of XIAP is accountable for binding and blocking the active sites of caspase-3 and caspase-7, and the BIR3 domain hinders the dimerization and activation of caspase-9 [11–14]. XIAP possesses an extra zinc-binding pattern known as the highly intriguing novel gene (RING) domain, which includes ubiquitin E3 ligase function that directs caspase-3 towards degradation and amplifies its anti-apoptotic impact [15]. In addition, the RING domain has the ability to modify XIAP itself and other targets that are likely involved in functions beyond cell death [16]. Nevertheless, limited information is available regarding the involvement of XIAP in the resistance of HCC cells to anoikis. The mitogen-activated protein kinases (MAPKs) can connect cell surface receptors to the regulatory targets in response to different stimuli, among which extracellular signal-regulated kinases (ERKs) function in controlling cell division [17]. Previous studies have shown that MAPK1 (ERK2) and MAPK3 (ERK1) are the essential components in the MAPK cascade and contribute to anoikis resistance in multiple cancers, including HCC [7, 18–20]. There is growing recognition that ERK1/2-dependent XIAP signaling is vital for the regulation of apoptosis [21–23].

This research aims to investigate the mechanism of XIAP underlying the resistance of HCC cells to anoikis. We observed that XIAP expression is positively associated with ERK1/2 expression in HCC tissues. Besides, our findings indicate that XIAP enhances the ability of HCC cells to resist anoikis. In addition, the BIR domains of XIAP play a crucial role in protecting against anoikis as well as the ERK1/2-mediated resistance of HCC cells to anoikis. Hence, targeting ERK1/2-dependent XIAP signaling may be a promising strategy to combat HCC.

## 2 | Results

### 2.1 | XIAP Expression Is Positively Correlated With ERK1/2 Expression in HCC

**FIGURE 1 |:**
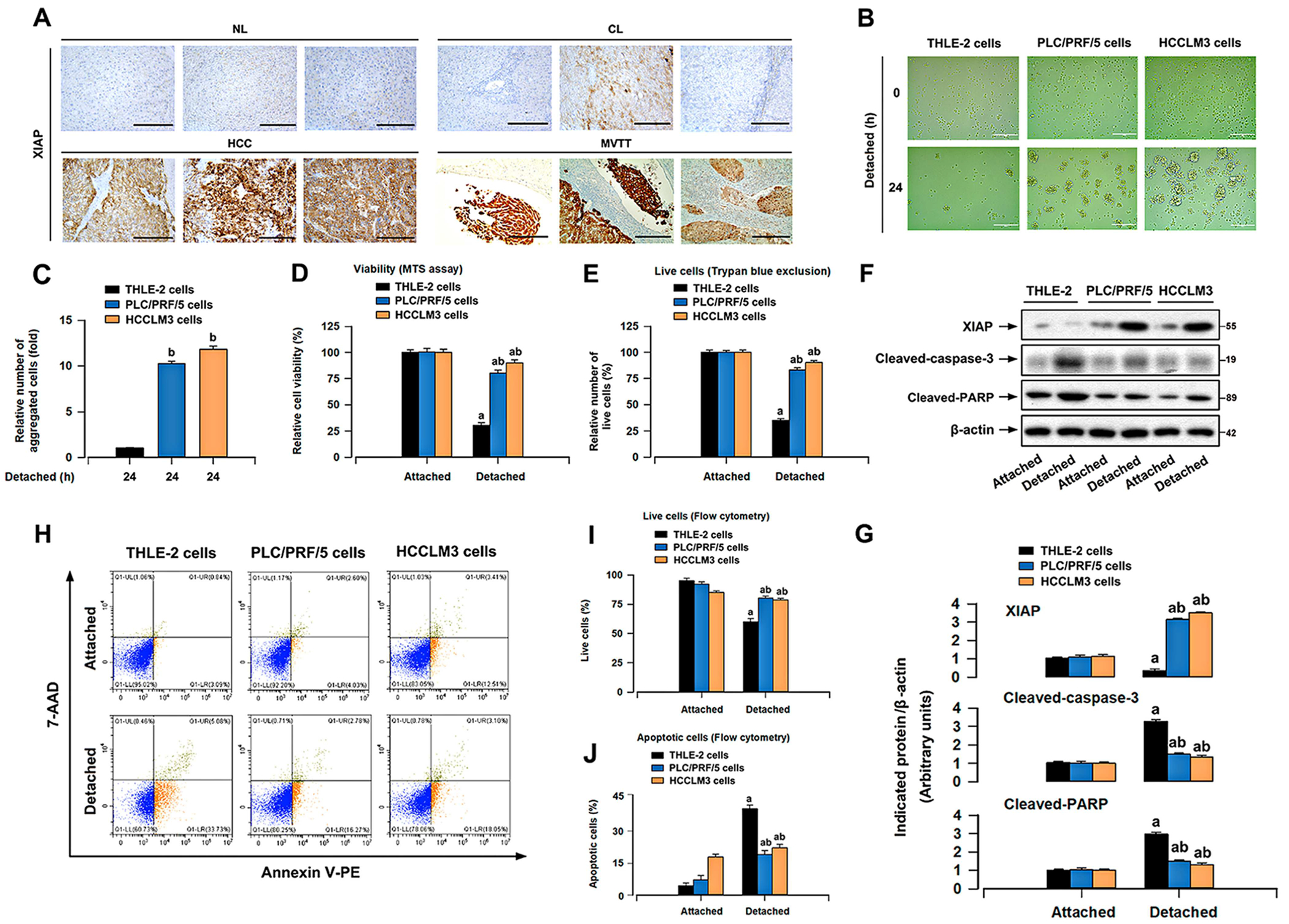
HCC cells exhibit a higher resistance to anoikis compared to normal hepatocytes. THLE-2, PLC/PRF/5, and HCCLM3 were cultured in normal plates to allow attachment for 24 h (for MTS assay, trypan blue exclusion test, and Annexin V-PE/7-AAD staining) or 12 h (for Western blotting), or transferred to ultra-low-attachment plates to induce detachment for 24 h or 12 h. (A) Representative pictures demonstrating XIAP staining in NL, CL, and HCC in situ/MVTT by IHC (scale bar: 200 μm). (B) The representative morphological images of cells cultured in detachment condition for 12 h (scale bar: 200 μm). (C) The relative number of aggregated colonies of cells in each field was counted after detachment. (D) The proportion of viable cells was quantified by MTS assay. (E) The relative number of live cells was determined by trypan blue exclusion test. (F) Western blot analysis of indicated proteins in cells. β-actin was probed as a loading control. (G) The relative density of XIAP, cleaved PARP, or cleaved caspase-3 was normalized against that of β-actin, with semi-quantitative analysis performed using NIH ImageJ software. (H) The proportions of viable (LL), dead (UL), early apoptotic (LR), and late apoptotic (UR) cells were quantified by FACS utilizing Annexin V-PE/7-AAD staining. (I, J) Quantitative analysis of live and apoptotic cells by FACS assay. All data were presented as mean ± SE, *n* = 3–5. ^a^*p* < 0.05, difference versus attached group; ^b^*p* < 0.05, difference versus detached THLE-2 cells group.

To determine the role of XIAP and ERK1/2 in HCC, relevant data were extracted from the Timer database based on The Cancer Genome Atlas (TCGA). The mRNA levels of XIAP and ERK1/2 were significantly increased in HCC in comparison to normal liver, as shown in Figure S1A. Similar results were verified in HCC from the GEPIA database, matched with TCGA normal and publicly available Genotype-Tissue Expression (GTEx) data (Figure S1B). Notably, by analysis of the Timer database, a positive linear association was identified between the expression levels of XIAP and ERK1/2 in HCC tissues (Figure S1C), which was also observed by analyzing the GEPIA database (Figure S1D). To validate the relationship between XIAP and ERK1/2 at the protein level, the IHC images of XIAP and ERK1/2 in HCC tissues and normal liver tissues were obtained from the Human Protein Atlas. The results revealed that both XIAP and ERK1/2 were upregulated in HCC in comparison to normal liver (Figure S1E). Importantly, the protein level of XIAP was positively associated with that of ERK2 in hepatocytes from Human Protein Atlas through the analysis of RNA single cell type specificity (Figure S1F). The ERK1/2 signaling has been reported to play a pivotal role in anoikis resistance of HCC cells [7]. In addition, HCC patients with high XIAP expression demonstrated shorter overall survival and disease-free survival (Figure S1G). To further examine the role of XIAP in human HCC progression, we evaluated the protein levels of XIAP in normal livers (NL), cirrhotic livers (CL), as well as HCC in situ and MVTT. Notably, the IHC analysis of liver samples verified the elevated XIAP expression in HCC, particularly in MVTT (Figure 1A).

### 2.2 | HCC Cells Are More Anoikis-Resistant Than Normal Hepatocyte Cells

When detached from matrix, cancer cells exhibit reduced sensitivity to anoikis in comparison to normal cells [24, 25]. In addition, the cells that make up multicellular aggregates have a higher resistance to anoikis compared to individual cells in suspension [25]. In this study, the THLE-2 cell line, an immortalized human normal liver cell, the PLC/PRF/5 cell line, having lower metastatic potential, and the HCCLM3 cell line, derived from an undifferentiated human HCC with strong metastatic potential [26], were employed to verify their sensitivity to anoikis. Consistently, PLC/PRF/5 and HCCLM3 cells formed larger colonies than THLE-2 cells in detached conditions (Figure 1B,C). Furthermore, we found that detachment reduced the viability profoundly in THLE-2 cells, but slightly in PLC/PRF/5 and HCCLM3 cells, as analyzed by MTS assay (Figure 1D). Comparable findings were obtained in the trypan blue exclusion test (Figure 1E). In addition, the enhanced levels of cleaved caspase-3/PARP, the markers of apoptosis, were more prominent in THLE-2 cells than in PLC/PRF/5 and HCCLM3 cells following detachment (Figure 1F,G). Similar results were observed in FACS using Annexin V-PE/7-AAD staining (Figure 1H–J). These observations in cell lines reveal that HCC cells are more anoikis-resistant.

### 2.3 | Detached HCC Cells Show an Increase in XIAP Expression

**FIGURE 2 |:**
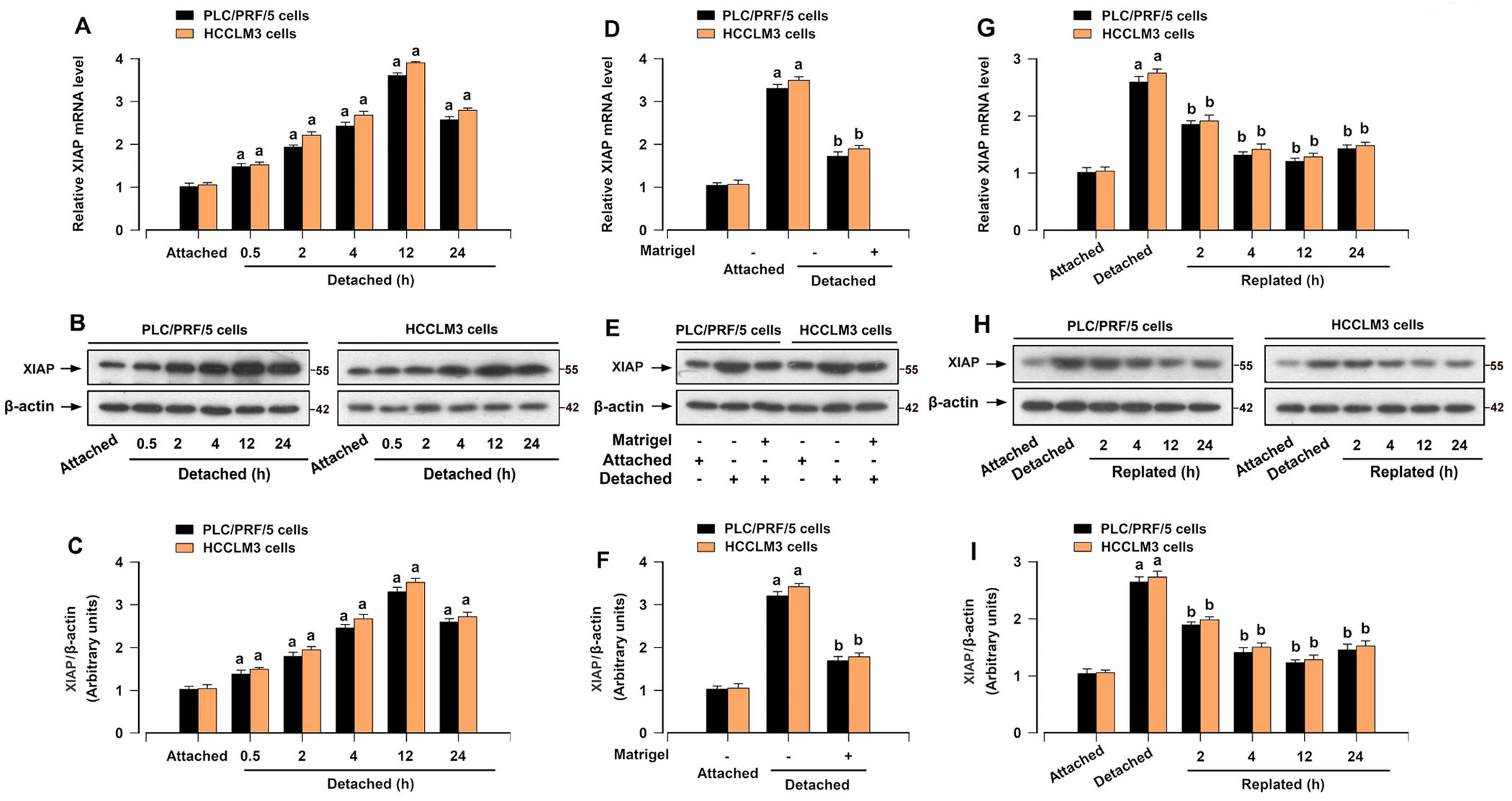
XIAP is upregulated in HCC cells after detachment. PLC/PRF/5 and HCCLM3 cells were attached for 12 h (for qRT-PCR and Western blotting), detached for the indicated time periods, pretreated with/without Matrigel followed by 12 h detachment, or detached for 12 h and then replated on normal plates for indicated time, respectively. (A, D, G) The mRNA level of XIAP was evaluated by qRT-PCR analysis. (B, E, H) Western blot analysis of indicated proteins in cells. β-actin was probed as a loading control. (C, F, I) The relative densities of XIAP normalized to β-actin in cellular lysates were subjected to semi-quantitative analysis utilizing NIH ImageJ. All data were presented as mean ± SE, *n* = 3–5. ^a^*p* < 0.05, difference versus attached group; ^b^*p* < 0.05, difference versus detached group.

XIAP is one of the most significant and powerful caspase inhibitors [27]. Next, we investigated the correlation between XIAP and resistance to anoikis in HCC cells. Significantly, the upregulation of XIAP due to detachment stimulation was more noticeable in HCCLM3 and PLC/PRF/5 cells (Figure 1F,G). To assess the involvement of XIAP in the resistance of HCC cells to anoikis, we further evaluated XIAP expression in HCC cells upon detachment by culturing cells for various times. XIAP mRNA and protein levels were elevated as early as 0.5 h following detachment, increased gradually and peaked at 12 h postdetachment, and sustained at high levels even at 24 h (Figure 2A–C). Given that the cell attachment is facilitated by a family of adhesion proteins called integrins [28], we then examined whether integrin signaling regulates the expression of XIAP. Addition of Matrigel to medium markedly mitigated the elevated mRNA and protein levels of XIAP in detached HCC cells (Figure 2D–F). Thus, the increase in XIAP expression upon detachment of HCC cells is, at least in part, due to the disruption of integrin signaling. Interestingly, we discovered that when HCC cells detached for 12 h were allowed to reattach, the levels of XIAP mRNA and protein were decreased (Figure 2G–I). The above results indicate a possible association between XIAP and anoikis resistance in HCC cells.

### 2.4 | XIAP Is Involved in Anoikis Resistance of HCC Cells

**FIGURE 3 |:**
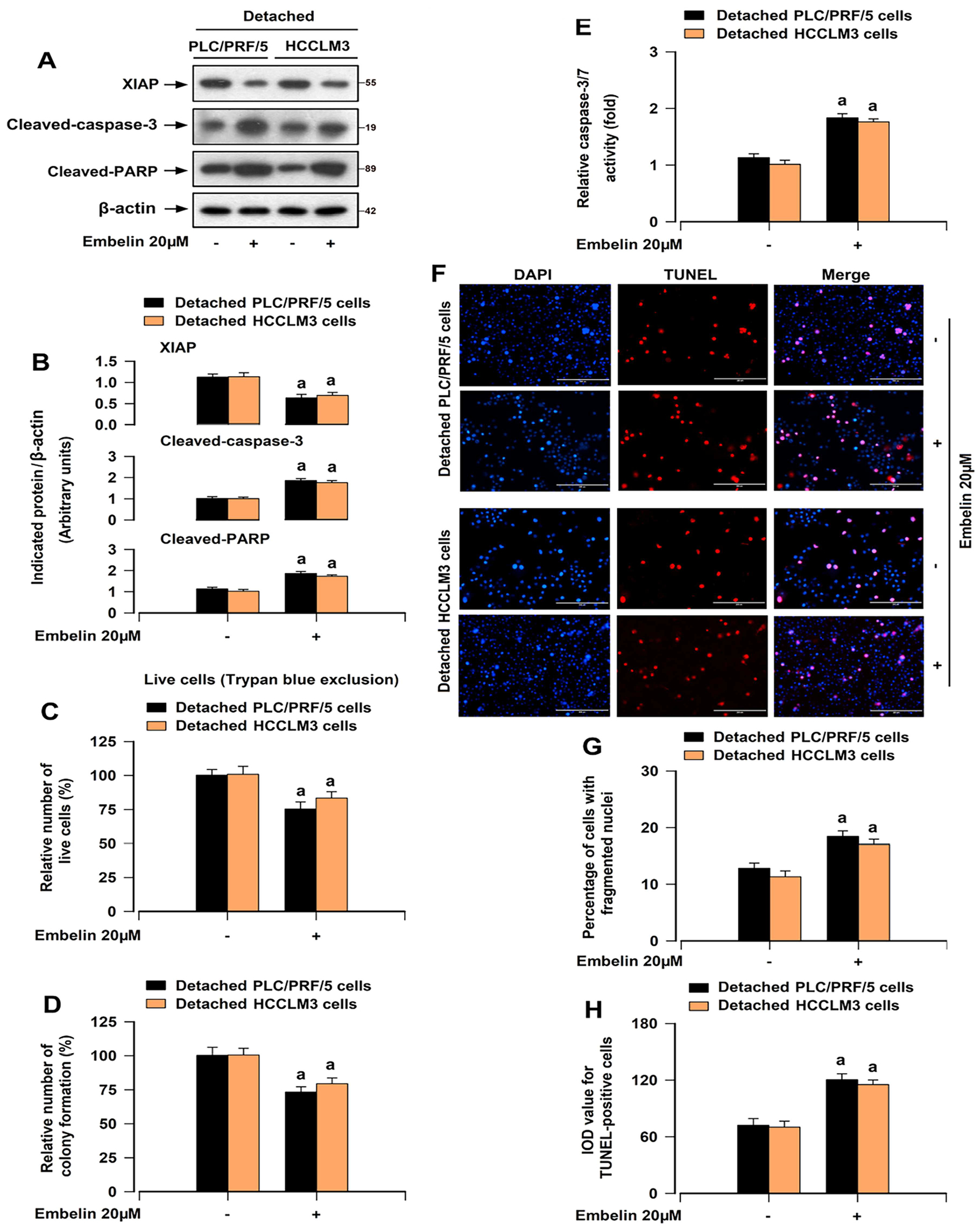
Pharmacological suppression of XIAP with Embelin induces anoikis of HCC cells. HCCLM3 and PLC/PRF/5 cells were detached for 12 h (for Western blotting), 24 h (for caspase-3/7 activity, DAPI/TUNEL staining, and trypan blue exclusion test), or 2 weeks (soft agar colony formation assay), with/without 1-h pretreatment with Embelin (20 μM). (A) Western blot analysis of indicated proteins in cells. β-actin was probed as a loading control. (B) The relative densities for XIAP, cleaved PARP, and cleaved caspase-3 to β-actin were semi-quantified using NIH ImageJ. (C) The relative number of live cells was determined by trypan blue exclusion test. (D) The ability for anchorage-independent growth was assessed by soft agar colony formation assay. (E) Caspase-3/7 activity was measured by Caspase-3/7 Assay Kit. (F) Apoptotic cells were identified by detecting nuclear fragmentation and condensation using DAPI staining and fragmented DNA by TUNEL staining (scale bar: 200 μm). (G) The percentage of cells with fragmented nuclei using DAPI staining was quantified. (H) The IOD value of TUNEL-positive cells with the fluorescence staining was quantified. All data were presented as mean ± SE, *n* = 3–5. ^a^*p* < 0.05, + Embelin group versus – Embelin group.

**FIGURE 4 |:**
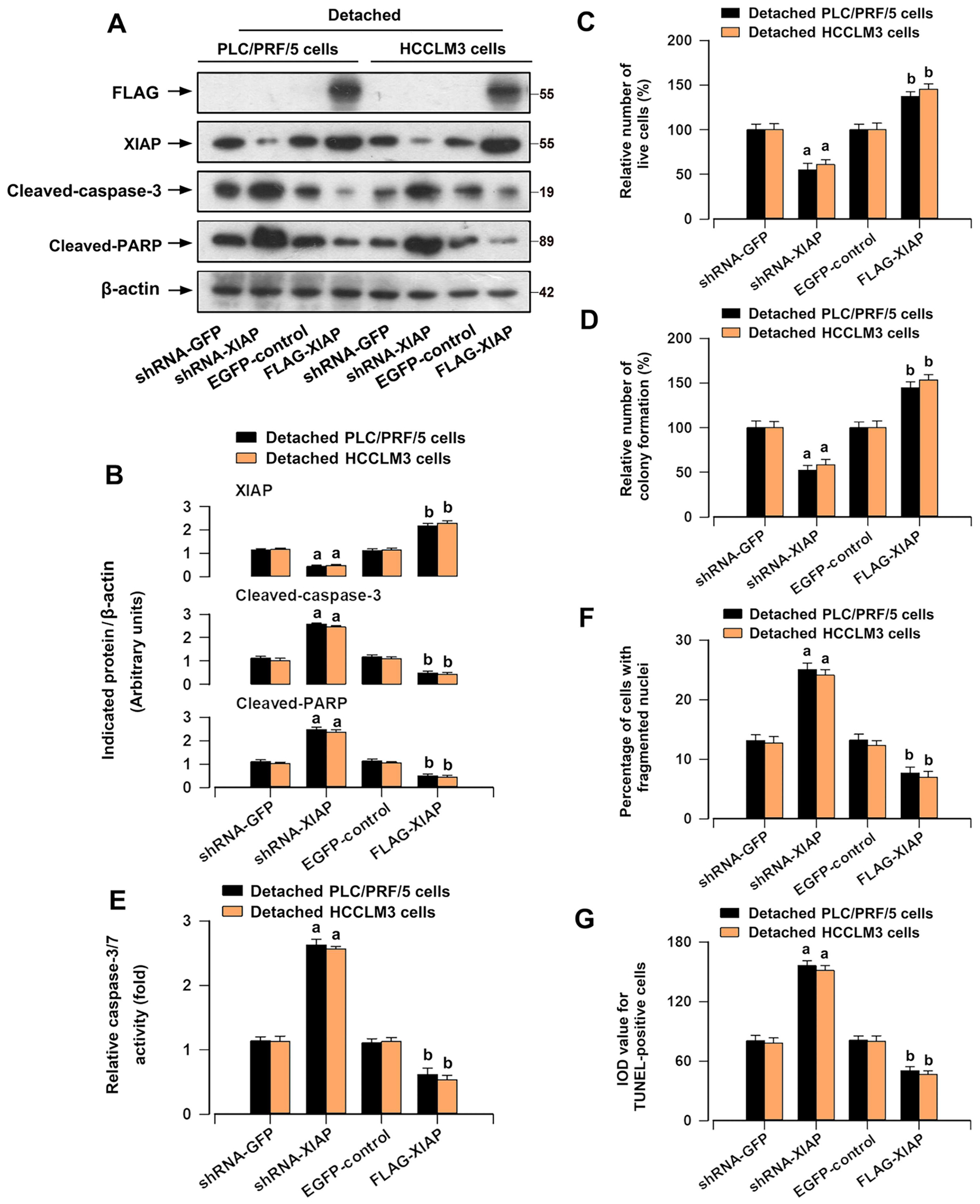
Downregulation or upregulation of XIAP affects anoikis resistance in HCC cells. PLC/PRF/5 and HCCLM3 cells infected with lentiviral shRNA to XIAP or GFP (as control), and lentiviral FLAG-tagged wild-type XIAP (FLAG-XIAP) or EGFP (as control), respectively, were detached for 12 h (for Western blotting), 24 h (for caspase-3/7 activity, DAPI/TUNEL staining, and trypan blue exclusion assay), or 2 weeks (soft agar colony formation assay). (A) Western blot analysis of indicated proteins in cells. β-actin was probed as a loading control. (B) The relative densities of XIAP, cleaved PARP, and cleaved caspase-3 to β-actin were subjected to semi-quantitative analysis utilizing NIH ImageJ. (C) The relative number of live cells was determined by trypan blue exclusion test. (D) The capability for anchorage-independent growth was assessed by soft agar colony formation assay. (E) Caspase-3/7 activity was evaluated by the Caspase-3/7 Kit. (F) The percentage of cells with fragmented nuclei using DAPI staining was quantified. (G) The IOD value of TUNEL-positive cells with the fluorescence staining was quantified. All data were presented as mean ± SE, *n* = 3–5. ^a^*p* < 0.05, shRNA-XIAP group versus shRNA-GFP group; ^b^*p* < 0.05, FLAG-XIAP group versus EGFP-control group.

To determine the involvement of XIAP in anoikis resistance of HCC cells, PLC/PRF/5 and HCCLM3 cells were treated with Embelin, an inhibitor that blocks XIAP binding to caspases [29]. XIAP expression was decreased in detached HCC cells under the treatment with Embelin (Figure 3A,B). Furthermore, Embelin reduced the survival and restrained anchorage-independent growth of HCC cells in response to detachment, as evidenced by trypan blue exclusion test and colony formation assay (Figure 3C,D). We also observed that pretreatment with Embelin increased the cleaved caspase-3/PARP expression (Figure 3A,B) and the caspases 3/7 activation in HCC cells following detachment (Figure 3E). Then, we used DAPI staining to investigate apoptosis of the HCC cells, which is characterized by nuclear fragmentation and condensation [30], and utilized TUNEL staining to identify the cells with fragmented DNA. The results obtained from imaging and quantification showed that Embelin elevated the proportion of the cells with nuclear fragmentation and condensation, and the number of TUNEL-positive cells with DNA fragmentation in detached HCC cells (Figure 3F–H). To corroborate the finding, XIAP was stably downregulated or upregulated in PLC/PRF/5 and HCCLM3 cells by infection with lentiviral shRNA-XIAP or lentiviral FLAG-XIAP (Figure 4A,B). We noted that downregulation or upregulation of XIAP induced or reduced the cleavages of caspase-3/PARP (Figure 4A,B) and caspases 3/7 activation (Figure 4E) in HCC cells after detachment. Knockdown of XIAP suppressed the survival and anchorage-independent growth of HCC cells in response to detachment (Figure 4C,D), but enhanced anoikis in detached HCC cells (Figure 4F,G). The reverse results were shown in XIAP-overexpressing HCC cells under detachment conditions (Figure 4C–G). Our results demonstrate that XIAP is vital for the ability of HCC cells to resist anoikis.

### 2.5 | The BIR Domains of XIAP Are Essential for Anoikis Resistance in HCC Cells

**FIGURE 5 |:**
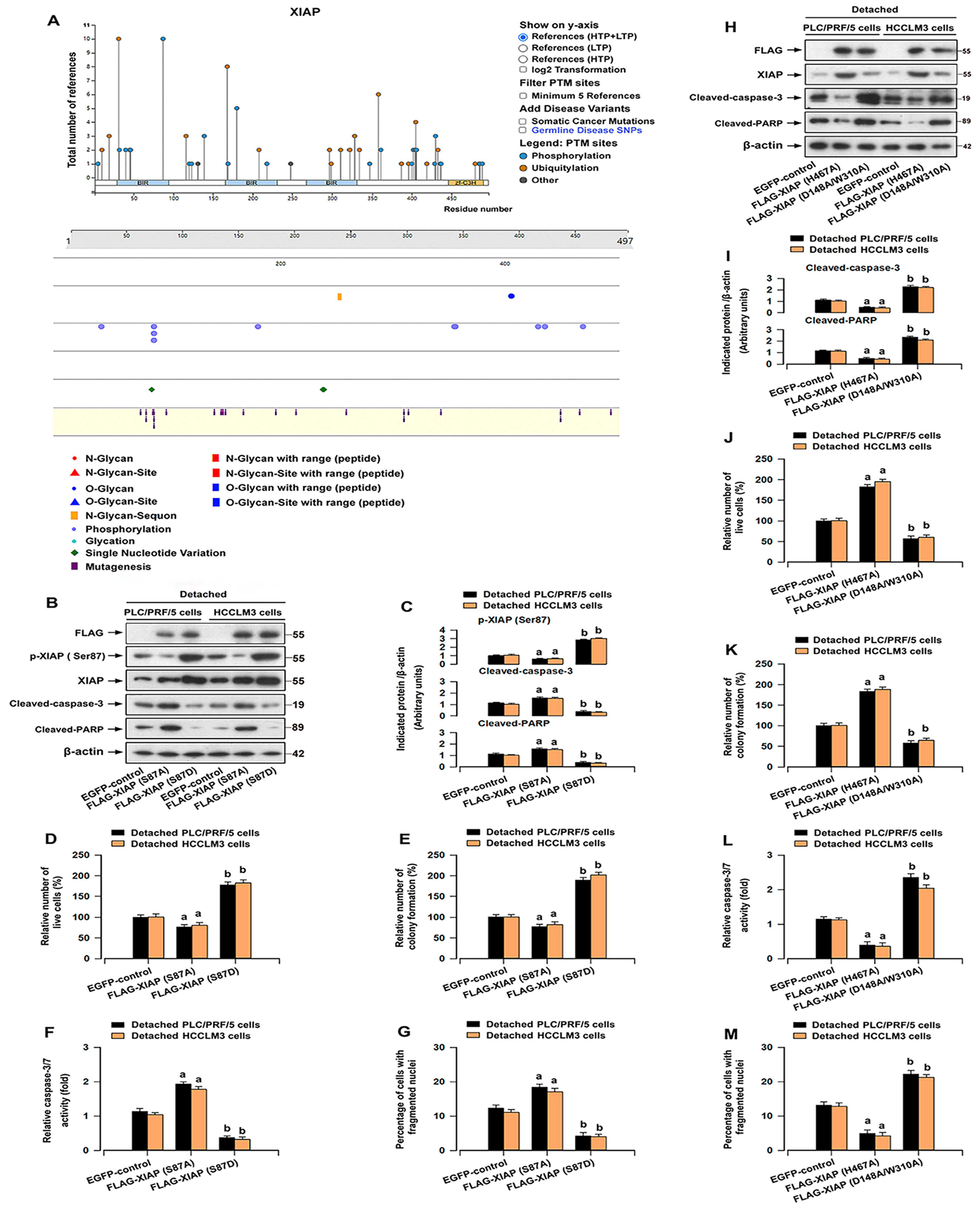
The BIR domains of XIAP enhance anoikis resistance in HCC cells. PLC/PRF/5 and HCCLM3 cells infected with lentiviral FLAG-XIAP (S87A), FLAG-XIAP (S87D), FLAG-XIAP (H467A), FLAG-XIAP (D148A/W310A), or EGFP (as control), respectively, were detached for 12 h (for Western blotting), 24 h (for caspase-3/7 activity, DAPI staining, and trypan blue exclusion test), or 2 weeks (soft agar colony formation assay). (A) Posttranslational modifications and mutagenesis of human XIAP protein from the PhosphoSitePlus () and GlyGen (). (B, H) Western blot analysis of indicated proteins in cells. β-actin was probed as a loading control. (C, I) The relative densities for p-XIAP (Ser87), XIAP, cleaved caspase-3, and cleaved PARP to β-actin were subjected to semi-quantitative analysis utilizing NIH ImageJ. (D, J) The relative number of live cells was determined by trypan blue exclusion test. (E, K) The capability for anchorage-independent growth was assessed by the soft agar colony formation assay. (F, L) Caspase-3/7 activity was determined using the Caspase-3/7 Assay Kit. (G, M) The proportion of cells with fragmented nuclei was quantified by DAPI staining. All data were presented as mean ± SE, *n* = 3–5. ^a^*p* < 0.05, FLAG-XIAP (S87A) group or FLAG-XIAP (H467A) group versus EGFP-control group; ^b^*p* < 0.05, FLAG-XIAP (S87D) group or FLAG-XIAP (D148A/W310A) group versus EGFP-control group.

The function of XIAP can be regulated by site phosphorylation or mutations, as identified by large-scale proteomic studies (Figure 5A). Although the functions of many of these sites remain unknown, it has been shown that increasing phosphorylation of XIAP on Ser87 protects ovarian cancer cells from apoptosis [31]. To evaluate whether the impact of this modification is involved in the resistance of HCC cells to anoikis, FLAG-tagged S87A (dominant-negative mutant) and S87D (constitutively active mutant) of XIAP were constructed using lentiviral technology. Besides, we also generated lentiviral vectors expressing other XIAP mutants, including FLAG-XIAP (H467A) (loss of the function of RING domain – E3 ubiquitin-ligase activity) [32] and FLAG-XIAP (D148A/W310A) (loss of the function of BIR 2/3 domains – caspase inhibitory activity) [33, 34]. As shown in Figure 5B,C,H,I, infection with lentiviral XIAP (S87D) or XIAP (H467A) increased the expression of XIAP, while infection with lentiviral XIAP (S87A) or XIAP (D148A/W310A) reduced the XIAP expression in detached HCC cells. Importantly, overexpression of mutant XIAP (S87D) or XIAP (H467A) increased the number of viable HCC cells under detachment conditions (Figure 5D,J) and the number of colony formation (Figure 5E,K). In addition, overexpression of XIAP (S87D) or XIAP (H467A) attenuated detachment-induced elevation of cleaved caspase-3/PARP (Figure 5B,C,H,I), as well as caspases 3/7 activation in HCC cells (Figure 5F,L). Consistently, overexpression of XIAP (S87D) or XIAP (H467A) resulted in decreased anoikis in HCC cells (Figure 5G,M). The opposite results were seen in detached HCC cells infected with lentiviral XIAP (S87A) or XIAP (D148A/W310A) (Figure 5B–M). These observations indicate that the BIR domains, rather than the RING domain, might be indispensable for maintaining anoikis resistance of HCC cells.

### 2.6 | XIAP Triggers Anoikis Resistance of HCC Cells in an ERK1/2-Dependent Manner

**FIGURE 6 |:**
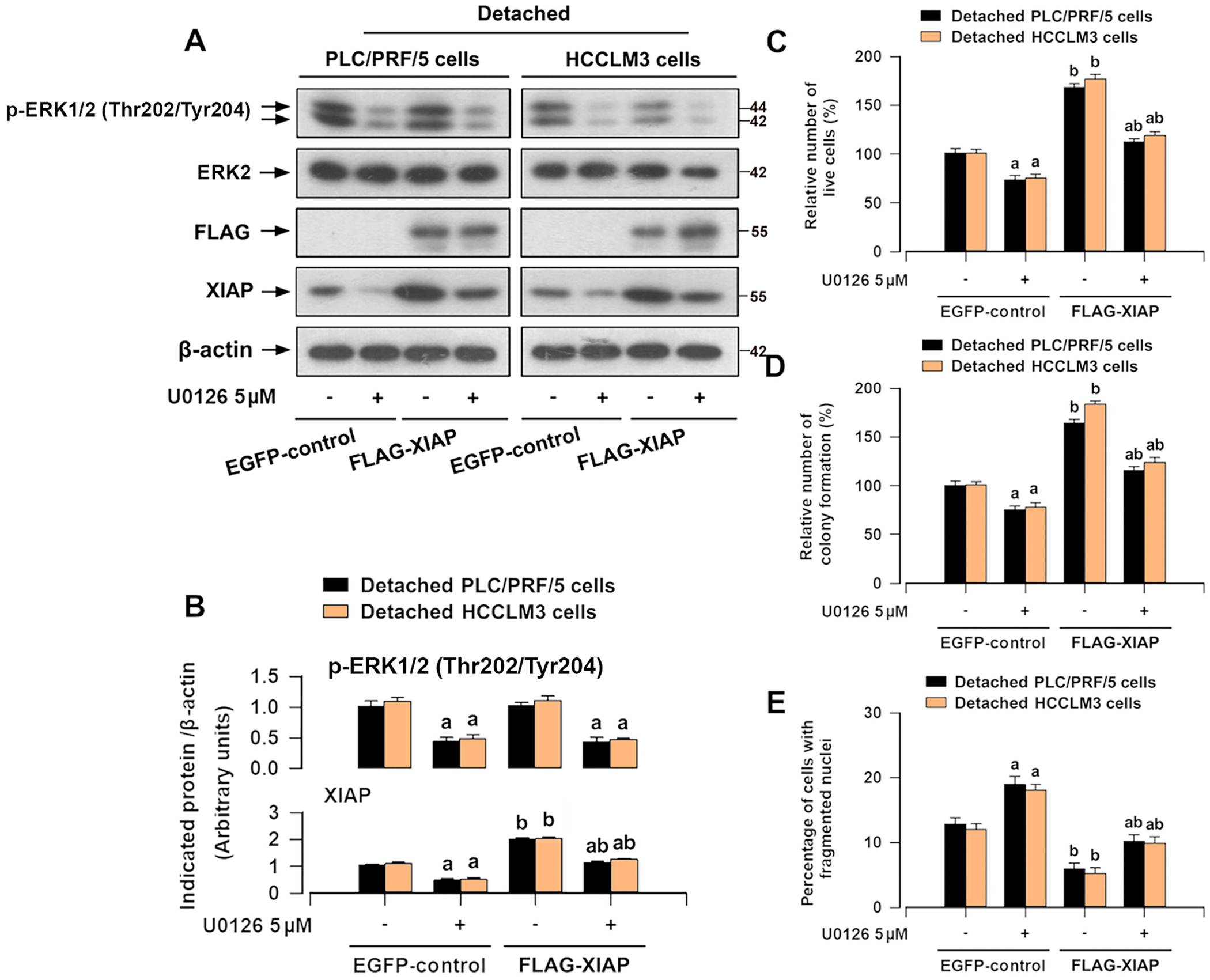
Inhibition of ERK1/2 by U0126 attenuates XIAP-evoked anoikis resistance of HCC cells. HCCLM3 and PLC/PRF/5 cells or XIAP-overexpressing HCCLM3 and PLC/PRF/5 cells, pretreated with/without U0126 (5 μM) at indicated concentrations for 1 h, and then detached for 12 h (for Western blotting), 24 h (for trypan blue exclusion assay and DAPI staining), or 2 weeks (soft agar colony formation assay). (A) Western blot analysis of indicated proteins in cells. β-actin was probed as a loading control. (B) The relative densities for p-ERK1/2 (Thr202/Tyr204) and XIAP to β-actin were subjected to semi-quantitative analysis utilizing NIH ImageJ. (C) The relative number of live cells was determined by trypan blue exclusion test. (D) The cell proliferation was estimated by the soft agar colony formation assay. (E) The percentage of cells with fragmented nuclei was quantified by DAPI staining. All data were presented as mean ± SE, *n* = 3–5. ^a^*p* < 0.05, + U0126 group versus − U0126 group; ^b^*p* < 0.05, FLAG-XIAP group versus EGFP-control group.

To determine whether XIAP participates in ERK1/2-dependent anoikis resistance of HCC cells, we employed U0126, a selective inhibitor of mitogen-activated protein kinase kinase 1/2 (MKK1/2), upstream of ERK1/2. We noticed that U0126 treatment inhibited the phosphorylation of ERK1/2 (Thr202/Tyr204) and XIAP expression in HCCLM3 and PLC/PRF/5 cells after detachment (Figure 6A,B). Besides, U0126 treatment decreased the viability (Figure 6C) and suppressed the anchorage-independent growth capability of detached HCC cells (Figure 6D). U0126 treatment increased anoikis in detached HCC cells (Figure 6E). Interestingly, U0126 treatment reduced the basal or lentiviral FLAG-XIAP-upregulated expression of XIAP, although overexpression of XIAP had no effect on ERK1/2 phosphorylation level in detached HCC cells (Figure 6A,B). Functional experiments showed that ERK1/2 inhibition compromised the protective effect of XIAP overexpression against anoikis of HCC cells (Figure 6C–E). The results suggest that ERK1/2 can positively regulate XIAP expression, causing anoikis resistance in HCC cells.

**FIGURE 7 |:**
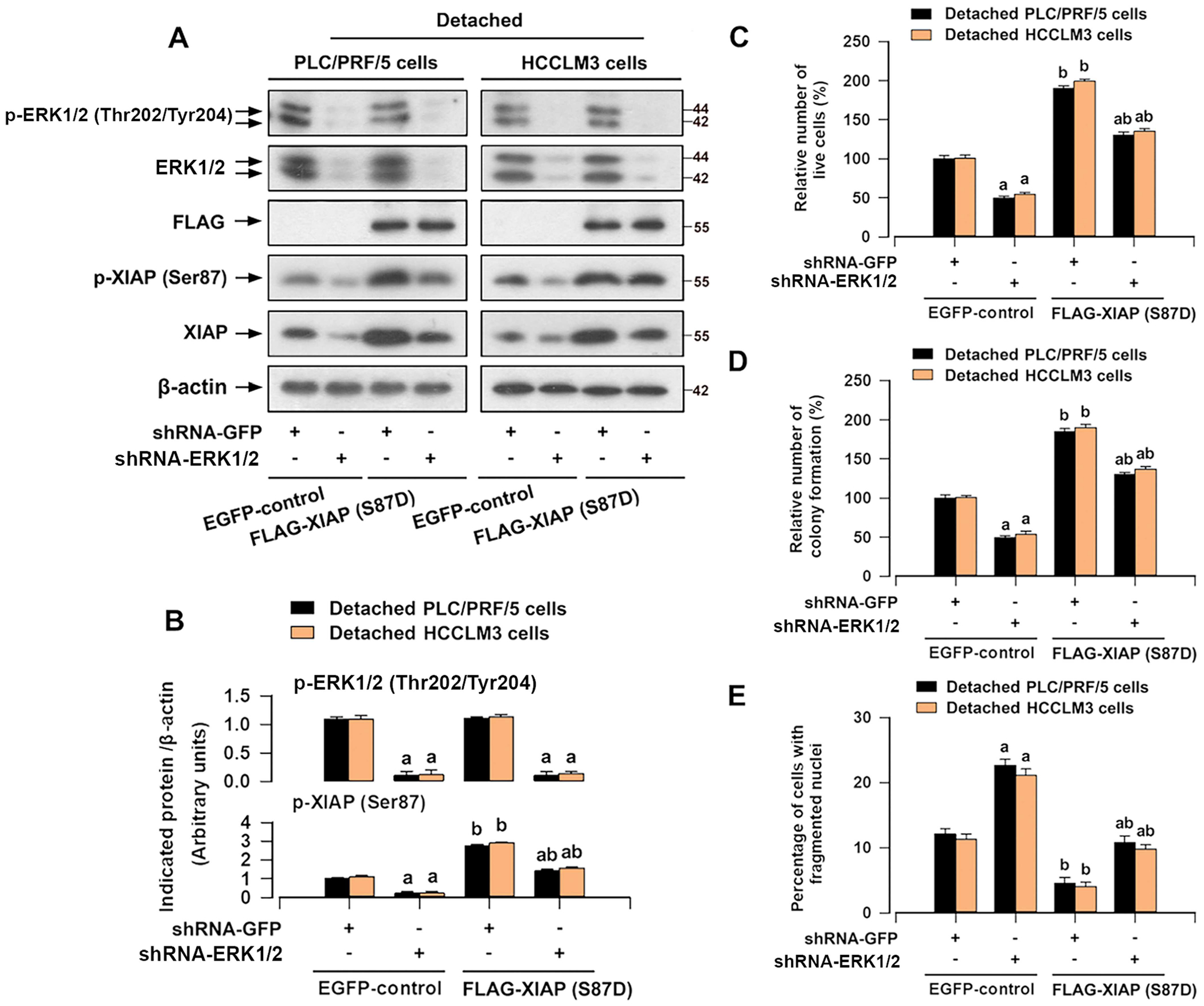
Overexpression of mutant XIAP (S87D) suppresses ERK1/2 deficiency-induced anoikis of HCC cells. HCCLM3 and PLC/PRF/5 cells or mutant XIAP (S87D)-overexpressing PLC/PRF/5 and HCCLM3 cells, infected with lentiviral shRNA to ERK1/2 or GFP (as control), respectively, were detached for 12 h (for Western blotting), 24 h (for trypan blue exclusion assay and DAPI staining), or 2 weeks (soft agar colony formation assay). (A) Western blot analysis of indicated proteins in cells. β-actin was probed as a loading control. (B) The relative densities for p- ERK1/2 (Thr202/Tyr204) and p-XIAP (Ser87) to β-actin were subjected to semi-quantitative analysis utilizing NIH ImageJ. (C) The relative number of live cells was evaluated by trypan blue exclusion test. (D) The cell proliferation was estimated by the soft agar colony formation assay. (E) The percentage of cells with fragmented nuclei was quantified by DAPI staining. All data were presented as mean ± SE, *n* = 3–5. ^a^*p* < 0.05, shRNA-ERK1/2 group versus shRNA-GFP group; ^b^*p* < 0.05, FLAG-XIAP (S87D) group versus EGFP-control group.

To further validate the pivotal role of ERK1/2 in XIAP-triggered anoikis resistance of HCC cells, we expanded our investigations through gene silencing experiment. Lentiviral shRNA to ERK1/2 successfully reduced the levels of ERK1/2 and p-ERK1/2 as well as the levels of XIAP and p-XIAP (Ser87) in detached HCC cells (Figure 7A,B). Knockdown of ERK1/2 induced significant anoikis of HCC cells (Figure 7C–E). As the phosphorylation of XIAP on Ser87 plays a vital role in its protein stability (Figure 5B,C), we speculated that ERK1/2 knockdown-induced downregulation of XIAP is associated with inhibition of p-XIAP (Ser87). For this, HCC cells were infected with lentiviral FLAG-XIAP (S87D). Overexpression of mutant FLAG-XIAP (S87D) did not affect the status of ERK1/2 or p-ERK1/2, but remarkably mitigated ERK1/2 knockdown-induced downregulation of XIAP and p-XIAP (Ser87) in HCC cells upon detachment (Figure 7A,B). Of note, overexpression of mutant FLAG-XIAP (S87D) abrogated ERK1/2 deficiency-induced anoikis of HCC cells (Figure 7C–E). Our results suggest that ERK1/2 lie upstream of XIAP, which can positively regulate the expression of XIAP, leading to anoikis resistance in HCC cells.

Furthermore, we utilized recombinant adenoviruses that contained FLAG-tagged dominant negative MKK1 (Ad-MKK1-K97M) and constitutively active MKK1 (Ad-MKK1-R4F), respectively. Infection of detached HCC cells with Ad-MKK1-K97M and Ad-MKK1-R4F led to high expression of FLAG-tagged MKK1 mutants (Figure S2A). MKK1-R4F expression induced strong expression of p-ERK1/2 (Thr202/Tyr204) and XIAP, whereas MKK1-K97M expression resulted in decreased levels of p-ERK1/2 (Thr202/Tyr204) and XIAP (Figure S2A,B), showing that the MKK1 mutants function as expected. However, silencing XIAP did not affect the status of p-ERK1/2 in HCC cells upon detachment (Figure S2A,B). Significantly, MKK1-R4F expression in detached HCC cells substantially increased the survival and anchorage-independent growth capability (Figure S2C,D), and conferred profound resistance to anoikis (Figure S2E), which was reversed by knockdown of XIAP (Figure S2C–E). In contrast, MKK1-K97M expression in detached HCC cells suppressed the survival and anchorage-independent growth capability (Figure S2C,D), and induced anoikis (Figure S2E), which was strengthened via knockdown of XIAP (Figure S2C–E). Collectively, these findings provide robust evidence for the concept that XIAP is a vital downstream molecule of MKK1-ERK1/2 in regulating HCC anoikis resistance.

### 2.7 | The BIR Domains of XIAP Are Required for ERK1/2-Mediated Anoikis Resistance in HCC Cells

The BIR domains and RING domain of XIAP have caspase inhibitory activity [11–14] and E3 ubiquitin-ligase activity [16], respectively. Next, we examined the involvement of these domains in ERK1/2-mediated anoikis resistance of HCC cells. HCC cells were infected with lentiviral XIAP (H467A, loss of the function of RING domain but having the intact function of BIR 1/2/3 domains) or XIAP (D148 A/W310A, loss of the function of BIR 2/3 domains but having the intact function of RING domain). There was no variation in ERK1/2 and p-ERK1/2 levels in detached HCC cells infected with lentiviral XIAP (H467A) or XIAP (D148 A/W310A) (Figure S3A,B). However, overexpression of mutant XIAP (H467A), which has no E3 ubiquitin-ligase activity [32], attenuated the downregulation of XIAP by ERK1/2 knockdown in HCC cells upon detachment (Figure S3A,B). Importantly, overexpression of mutant XIAP (H467A) alleviated ERK1/2 knockdown-induced anoikis, evidenced by reduced cell viability (Figure S3C), decreased colony formation (Figure S3D), and increased DNA fragmentation (Figure S3E). In contrast, overexpression of mutant XIAP (D148 A/W310A) strengthened ERK1/2 knockdown-induced anoikis in HCC cells following detachment (Figure S3A–E). These results suggest that BIR domains of XIAP, instead of RING domain, participated in ERK1/2-mediated anoikis resistance in HCC cells.

### 2.8 | XIAP Is Critical in ERK1/2-Mediated HCC Anoikis Resistance and Intrahepatic Spread in Mouse Models

**FIGURE 8 |:**
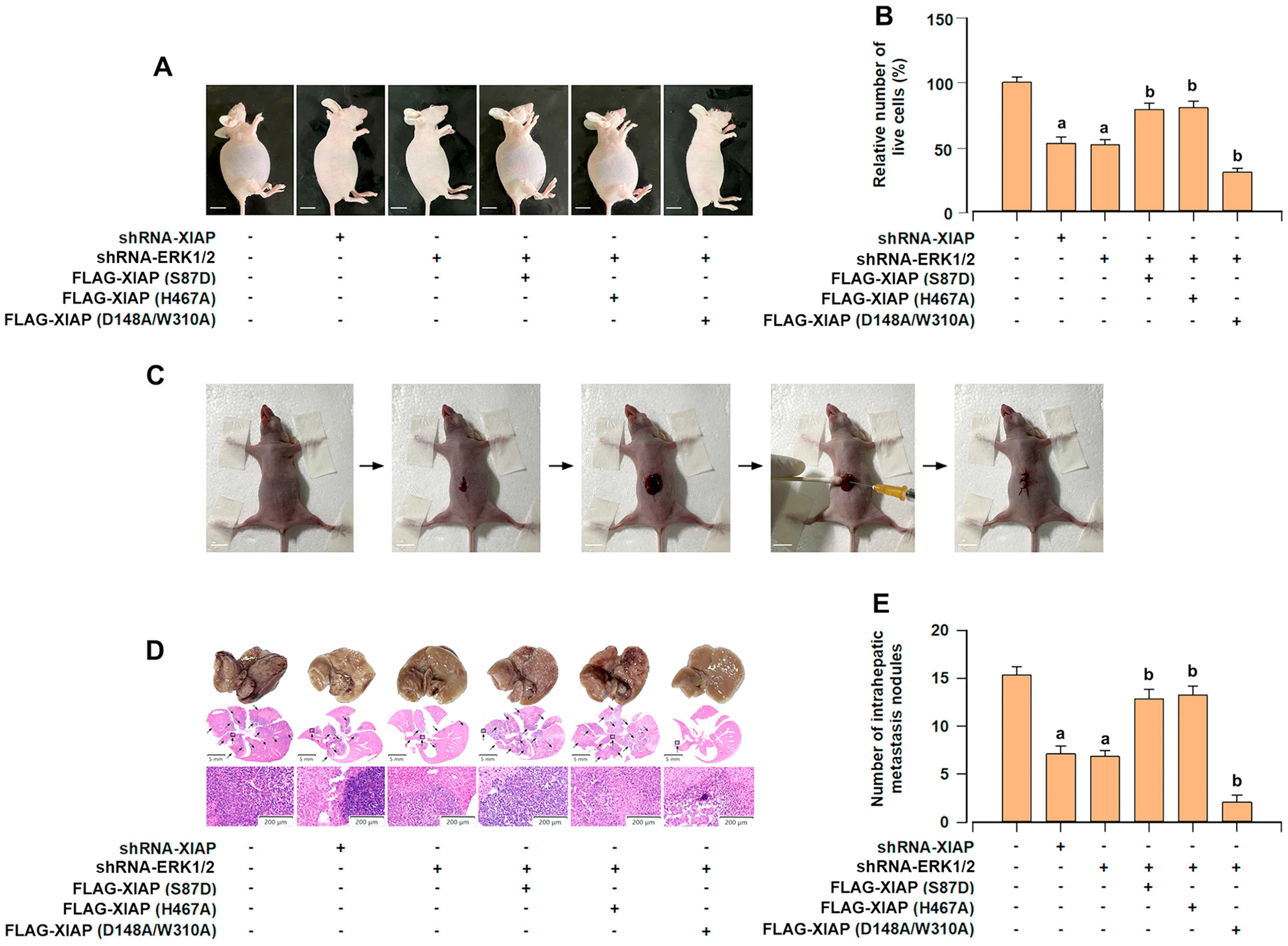
ERK1/2-mediated expression of XIAP controls anoikis resistance and intrahepatic metastasis *in vivo*. HCCLM3 cells, XIAP-deficient HCCLM3 cells, ERK1/2-deficient HCCLM3 cells, and ERK1/2-deficient HCCLM3 cells infected with lentiviral FLAG-XIAP (S87D), FLAG-XIAP (H467A), or FLAG-XIAP (D148A/W3110A), respectively, were intraperitoneally injected into BALB/c nude mice (for peritoneal cavities model) or intrahepatically injected into BALB/c nude mice (for hepatic orthotopic transplantation metastatic model). (A) The representative photographs of mouse peritoneal cavity model from the indicated groups obtained 7 days after intraperitoneal injection (scale bar: 10 mm). (B) HCCLM3 cells were collected from ascites, and the live cells were evaluated by trypan blue exclusion test. (C) The procedure for orthotopic implantation of indicated HCCLM3 cells into the liver parenchyma of mouse (scale bar: 10 mm). (D) The typical pictures of liver tissues and H&E staining in the different groups 4 weeks after orthotopic implantation with indicated HCCLM3 cells. The arrows indicate visible intrahepatic metastatic tumors. (E) The intrahepatic metastatic nodules from mouse orthotopic implantation model were quantified. All data were presented as mean ± SE, *n* = 3–5. ^a^*p* < 0.05, difference versus control group; ^b^*p* < 0.05, difference versus shRNA-ERK1/2 group.

To evaluate the in vivo role of XIAP in ERK1/2-mediated resistance to anoikis in HCC, we employed HCCLM3 cells in a mouse peritoneal cavity model. For this, HCCLM3 cells were intraperitoneally injected into BALB/c nude mice. Aligned with the in vitro results, knockdown of XIAP restrained the amount of peritoneal ascites and the viability of HCC cells (Figure 8A,B). Besides, ERK1/2-deficient HCCLM3 cells displayed a significant decrease in peritoneal ascites and HCC cell number, while these effects were reversed by overexpression of mutant FLAG-XIAP (S87D) or mutant XIAP (H467A) (Figure 8A,B). In contrast, overexpression of mutant XIAP (D148A/W310A) strengthened these inhibitory effects (Figure 8A,B).

To further confirm whether ERK1/2-mediated XIAP regulates HCC intrahepatic spread in vivo, we established the orthotopic xenograft model by injecting indicated HCCLM3 cells into the left lobe of the liver in immunodeficient nude mice (Figure 7C). In vivo assays showed that silencing XIAP or ERK1/2 reduced the number of intrahepatic tumors in mice (Figure 8D,E). In addition, overexpression of mutant FLAG-XIAP (S87D) or mutant XIAP (H467A) reversed the inhibition of intrahepatic spread caused by depletion of ERK1/2 (Figure 8D,E), while overexpression of mutant XIAP (D148A/W310A) further suppressed intrahepatic tumors (Figure 8D,E). Collectively, our data indicate that the BIR domains of XIAP are vital for ERK1/2-mediated anoikis resistance and intrahepatic spread of HCC cells in vivo.

## 3 | Discussion

The intricate molecular mechanisms of cancer cell resistance to anoikis are multifaceted [20]. XIAP is a multi-functional protein that regulates many cellular functions of cancer [35, 36]. Earlier studies have shown that human HCC cells exhibit elevated levels of IAPs [37], and enhanced XIAP expression in HCC contributes to the metastasis of tumor [38]. This prompted us to study whether and how XIAP protects HCC cells from anoikis. ERK1/2 signaling, among various oncogenic biomarkers, is markedly activated and plays a critical role in anoikis resistance of HCC cells [7]. Based on the bioinformatics analysis, we found a significant positive linear correlation between XIAP expression and ERK1/2 expression in HCC tissues (Figure S1A–F). The IHC staining of the array of liver samples confirmed the high expression of XIAP in HCC, particularly in MVTT (Figure 1A), suggesting that XIAP might be associated with anoikis resistance in HCC.

Cancer cells exhibit reduced sensitivity to anoikis in comparison to normal cells [24, 25]. During the progression of suspension culture, HCC cells, particularly those with high metastatic potential, exhibited greater resistance to anoikis compared to normal hepatocyte cells (Figure 1). Obviously, detachment-stimulated upregulation of XIAP was more prominent in HCC cells (Figure 1F,G). In addition, the increase in XIAP expression upon detachment of HCC cells is at least partially attributed to the disruption of integrin signaling (Figure 2). Further exploration is needed for these components of the mechanism. Subsequently, we found that inhibition of XIAP by Embelin compromised the capacity of anchorage-independent growth and anoikis resistance in HCC cells (Figure 3). Moreover, XIAP knockdown enhanced anoikis in HCC cells, while XIAP overexpression had the opposite effect (Figure 4). These results indicated that upregulation of XIAP in HCC increases anoikis resistance. Although our knowledge of XIAP structure and its molecular interactions with substrates has been advanced, the regulation of XIAP function in HCC cells remains limited. It has been shown that phosphorylation of XIAP on Ser87 stabilizes XIAP and protects ovarian cancer cells from apoptosis [31]. Our results further supported that the phosphorylation of Ser87 was required for stabilization of XIAP (Figure 5B,C) and that this function contributed to anoikis resistance in HCC cells (Figure 5B–G). Furthermore, we found that overexpression of mutant XIAP (H467A), which has no function of the RING domain but maintains the function of BIR domains of XIAP [32], inhibited anoikis in detached HCC cells (Figure 5H–M). Conversely, overexpression of mutant XIAP (D148A/W310A), which has no function of BIR 2/3 domains but maintains the function of RING domain of XIAP [33, 34], strengthened anoikis in HCC cells following detachment (Figure 5H–M). These findings indicate that the BIR domains, instead of RING domain, play an indispensable role in maintaining anoikis resistance of HCC cells.

It is increasingly evident that crosstalk between XIAP and ERK1/2 is important for apoptosis or survival, best exemplified by the regulation of XIAP expression via ERK1/2 signaling [21–23] or ERK1/2 activity regulated by the RING domain of XIAP [39]. Our results indicated that inactivation of ERK1/2 using its inhibitor U0126 compromised the protective effects of XIAP overexpression against anoikis of HCC cells (Figure 6). In addition, overexpression of mutant FLAG-XIAP (S87D) abrogated ERK1/2 deficiency-induced anoikis of HCC cells (Figure 7), hence further supporting the notion that XIAP-induced anoikis resistance is regulated by ERK1/2 in HCC cells. ERK1/2 may regulate the phosphorylation of XIAP (Ser87), stabilizing XIAP protein in HCC cells. In addition, as illustrated in Figure 2, detachment clearly increased XIAP mRNA expression, suggesting that XIAP alteration occurs at the transcriptional level as well. Indeed, it is well established that ERK1/2 can promote the activation of NF-κB [40]. Zhan et al. demonstrated that NF-κB p65 overexpression results in a significant increase in XIAP mRNA expression [41]. Possibly, ERK1/2 may regulate the transcriptional levels of XIAP by activating NF-κB. Given that XIAP (Ser87) is associated with the stability of the XIAP protein [42, 43], we propose that ERK1/2 signaling activation may not only enhance XIAP mRNA expression but also stabilize XIAP protein through phosphorylation.

The BIR domains and RING domain of XIAP have caspase inhibitory activity [11–14] and E3 ubiquitin-ligase activity [16], respectively. Next, we examined which domain of XIAP is involved in ERK1/2-mediated anoikis resistance of HCC cells. Significantly, our research revealed that the BIR domains of XIAP were required for ERK1/2-mediated anoikis resistance in HCC cells (Figure S3).

MKK1/2 is a dual-specificity kinase known for activating ERK1/2 in cancer [44]. We further validate the vital role of MKK1 in XIAP-triggered anoikis resistance of HCC cells. Notably, overexpression of constitutively active MKK1 in detached HCC cells remarkably elevated the survival and anchorage-independent growth capability (Figure S2C,D), and conferred profound resistance to anoikis (Figure S2E), which was reversed via knockdown of XIAP (Figure S2C–E). On the contrary, overexpression of dominant negative MKK1 in detached HCC cells repressed the survival and anchorage-independent growth capability (Figure S2C,D), and induced anoikis (Figure S2E), which was strengthened by knockdown of XIAP (Figure S2C–E). Finally, in vivo effects of XIAP on ERK1/2-mediated anoikis resistance and intrahepatic metastasis (Figure 8) also supported our in vitro experimental findings. Collectively, these data indicated that XIAP is a pivotal downstream effector of the MKK1-ERK1/2 pathway and promotes anoikis resistance in HCC cells.

There are still some limitations in our study. First, our study preliminarily revealed the correlation between XIAP and ERK1/2 at mRNA and protein levels. However, p-ERK1/2 is crucial for XIAP expression. Activated ERK1/2 may regulate the phosphorylation of XIAP (Ser87), stabilizing XIAP protein in HCC cells, which cannot be reflected in analyses of public databases. Second, more liver cancer cell lines, such as HepG2 and Huh7, are required to enhance the generalizability of our conclusion. Third, this study only focuses on the regulatory mechanism of ERK1/2-XIAP signaling in anoikis resistance. Other relevant pathway proteins need to be tested to rule out cross-effects.

## 4 | Conclusion

In summary, the current research has shown that XIAP functions as a cancer promoter and is positively regulated by ERK1/2 signaling to execute its anoikis resistance effect in HCC. Importantly, increased phosphorylation of XIAP at Ser87 promotes HCC anoikis resistance. Furthermore, the BIR domains of XIAP are essential for ERK1/2-mediated anoikis resistance in HCC. Targeting ERK1/2-dependent XIAP signaling may be a promising strategy to combat HCC. Further research in this area could lead to the development of novel and targeted therapies for HCC and other cancers.

## 5 | Methods and Materials

### 5.1 | Data Collection

The TIMER database (https://cistrome.shinyapps.io/timer/) and GEPIA database (http://gepia.cancer-pku.cn/) were used to determine the role of XIAP and ERK1/2 in HCC. All data were downloaded prior to October 14, 2024, with no access or usage restrictions applicable during this period. The Human Protein Atlas database (https://www.proteinatlas.org) was used to validate the relationship between XIAP and ERK1/2 at the protein level. All data were downloaded prior to October 14, 2024, without controlled-access requirement.

### 5.2 | Cell Culture and In Vitro Anoikis Assay

THLE-2 and PLC/PRF/5 cells were procured from the American Type Culture Collection (ATCC) (Manassas, VA, USA). HCCLM3 cells were obtained from the Liver Cancer Institute (Fudan University, Shanghai, China) [45]. THLE-2 cells were cultured using the BEGM BulletKit (Lonza, Basel, Switzerland). PLC/PRF/5 and HCCLM3 cells were cultivated in Dulbecco’s Modified Eagle’s medium (DMEM), with the incorporation of 10% fetal bovine serum (FBS). The cells were then subjected to incubation in a humidified incubator at 37°C with 5% CO_2_. Anoikis was induced by culturing cells in ultra-low-attachment plates (Cat. #7007, Corning, NY, USA) [46]. To restore integrin signaling in detached HCC cells, the medium was supplemented with Matrigel (Cat. #354234, Corning, NY, USA) [6].

### 5.3 | Apoptotic Cell Detection by Flow Cytometry

The cells were planted in six-well normal or ultra-low-attachment plates at a concentration of 5 × 10^5^ cells/well. In accordance with the prescribed protocol, the proportions of viable, dead, early apoptotic, and late apoptotic cells were observed using a FACS Vantage SE flow cytometer (Becton Dickinson, CA, USA) with the Annexin V-PE/7-AAD Apoptosis Kit (Cat. #A213-02, Vazyme, Nanjing, China).

### 5.4 | Anchorage-Independent Growth by Colony Formation

The indicated PLC/PRF/5 and HCCLM3 cells, suspended in DMEM supplemented with 1% FBS and 0.35% agar, were seeded at a concentration of 5 × 10^3^ cells per well onto a 0.6% agar layer in six-well plates. The agars were nourished with the specified culture medium every 3 days. After 2-week culture, the visible colonies were observed using a light microscope (200×) (Leica DMi8, Wetzlar, Germany) and analyzed with NIH ImageJ software (National Institutes of Health, Bethesda, MD, USA).

### 5.5 | Lentiviral Cloning and Production

For the generation of lentiviral shRNA to XIAP, oligonucleotides including the target sequences underwent synthesis and annealing procedures sequentially. These processed fragments were subsequently inserted into the FSIPPW lentiviral vector by means of the EcoR1/BamH1 restriction enzyme site [47]. To create FLAG-tagged mutant constructs of XIAP (S87A), XIAP (S87D), XIAP (H467A), and XIAP (D148A/W310A), site-directed mutagenesis was performed following the description [48]. The sequences of oligonucleotides and primers utilized in the experiments are listed in Table S2. Then, the PCR products of FLAG-XIAP (S87A), FLAG-XIAP (S87D), FLAG-XIAP (H467A), and FLAG-XIAP (D148A/W310A) were cloned into pSin4-EF2-IRES-Pur vector by EcoRI/BamHI double-digestion.

For lentivirus production, the plasmids constructed as described above were co-transfected into 293TD cells along with pMD2.G and psPAX2 (both obtained from Addgene, Watertown, MA, USA), with the transfection process mediated by MegaTran 1.0 reagent (Cat. #TT200003, OriGene Technologies, Rockville, MD, USA). The supernatants including viral particles were gathered after 48 and 60 h post-transfection, pooled, filtered through polyethersulfone (PES) filter, aliquoted, and preserved at −80°C for experimental utilization. Lentiviral shRNAs to ERK1/2 and green fluorescent protein (GFP), EGFP, and FLAG-tagged wild-type XIAP (FLAG-XIAP) were generated and utilized as previously described [49, 50].

### 5.6 | Recombinant Adenoviral Constructs

To generate recombinant adenoviruses that express FLAG-tagged constitutively active as well as dominant negative MKK1, we excised DNA fragments encoding the relevant mutants from pMCL-MKK1-K97M and pMCL-MKK1-R4F. The isolated fragments were subsequently subcloned into the FLAG-tagged pENTR11 shuttle vector to enable subsequent experimental processes. The construction of the recombinant adenovirus was performed utilizing ViraPower Adenoviral Gateway Expression Kit (Cat. #K493000, Invitrogen, Carlsbad, CA, USA), with all procedures strictly following the protocols specified by the manufacturer. The recombinant adenovirus expressing GFP (Ad-GFP) was procured from GENECHEM Co. Ltd. (Shanghai, China).

### 5.7 | In Vivo Anoikis Experiments

Female BALB/c nude mice (6 weeks old; 6 mice per group) were purchased from Jiangsu Huachuang Sino Pharmaceutical Technology Corporation (Taizhou, China). All experimental mice were bred under SPF conditions at Nanjing Normal University, which maintained a 12-h light/dark cycle and controlled the environment at a constant temperature of 22°C ± 0.5°C. The animal procedures were executed in strict conformity with the regulations stipulated in the Guide for the Care and Use of Laboratory Animals. All animals always had ad libitum access to standard rodent feed and sterilized drinking water, except during designated experimental protocols. To confirm the in vivo anoikis assay, a model of peritoneal dissemination in mice was utilized [6]. HCCLM3 cells (1 × 10^6^ cells/100 μL) infected with the indicated lentivirus were intraperitoneally injected into mice. After treatment for 7 days, GFP-positive cells were obtained for trypan blue exclusion. For orthotopic xenograft model, HCCLM3 cells (1 × 10^6^ cells/100 μL) infected with the indicated lentivirus were resuspended in 50 μL mixture containing phosphate-buffered saline (PBS) and Matrigel (1:1). This mixture was then injected into the liver through a 10-mm incision in epigastrium under pentobarbital anesthesia. After 8 weeks, mice were euthanized to examine the intrahepatic tumor. The livers were stained with hematoxylin and eosin (H&E).

### 5.8 | Statistical Analysis

All experimental data were presented as mean ± standard error (SE). Three or more independent biological replicates were conducted for each experiment. For comparisons between two independent groups, the non-paired Studenťs *t*-test was employed to evaluate the statistical significance of observed differences. One-way or two-way ANOVA was utilized to compare group variability and interactions, whilst Bonferroni’s post-tests were employed to assess replicate means. Statistical analyses employed GraphPad Prism 8 (USA), with *p* < 0.05 establishing significance.

## Supplementary Material

Supplementary Material

Additional supporting information can be found online in the Supporting Information section.

3 Re-revised Supporting Information-011526.

## Data Availability

The data used to support the findings of this study are available from the corresponding author upon reasonable request.
