# Supplementary material for: X-Linked Inhibitor of Apoptosis Protein (XIAP) Contributes to ERK1/2-Mediated Anoikis Resistance in Hepatocellular Carcinoma
Source: MedComm Oncol. Author manuscript; Available in PMC 2026 May 5. (PMC13137156; doi:10.1002/mog2.70059)
Supplement: Supplementary Material [file NIHMS2165691-supplement-Supplementary_Material.doc]

**X-linked inhibitor of apoptosis protein (XIAP) contributes to ERK1/2-mediated anoikis resistance in hepatocellular carcinoma**

Qingyu Zeng^1,2#^, Zun Mao^1,3,#^, Sumin Sun^1^, Zhixiang Gao^1^, Junpeng Mu^4^, Zhaoji Pan^5^, Qing Li^6^, Xueyuan Mao^7^, Jianbo Xu^8^, Dousheng Bai^3^, Shile Huang^9,10,11**^, Long Chen^1,*^

**Affiliations**

^1^ Jiangsu Key Laboratory for Molecular and Medical Biotechnology, College of Life Sciences, Nanjing Normal University, Nanjing 210023, PR China

^2^ Institute of Photomedicine, Shanghai Skin Disease Hospital, School of Medicine, Tongji University, Shanghai 200092, China

^3^ Department of Hepatobiliary Surgery, Northern Jiangsu People's Hospital, Yangzhou 225003, PR China.

^4^ Research Institute of General Surgery, Jinling Hospital, School of Medicine, Nanjing University, Nanjing 210093, PR China

^5^ Clinical Laboratory, Xuzhou Central Hospital, Southeast University Affiliated Xuzhou Central Hospital, Xuzhou 221000, PR China

^6^ Department of Pathology, Xuzhou Central Hospital, Xuzhou Clinical School of Xuzhou Medical University, Xuzhou 221000, PR China

^7^ Department of Pathology, The Suqian Clinical College of Xuzhou Medical University, Suqian 223800, PR China

^8^ Department of Hepatobiliary Surgery, The Affiliated Huaian No. 1 People's Hospital of Nanjing Medical University, Huai’an 223001, PR China

^9^ Department of Biochemistry and Molecular Biology, Louisiana State University Health Sciences Center, Shreveport, LA 71103, USA

^10^ Department of Hematology and Oncology, Louisiana State University Health Sciences Center, Shreveport, LA 71103, USA

^11^ Feist-Weiller Cancer Center, Louisiana State University Health Sciences Center, Shreveport, LA 71103, USA

^#^These authors contributed equally.

**Correspondence**

**^*^** Long Chen, Ph.D. (College of Life Sciences, Nanjing Normal University, 1 Wenyuan Road, Chixia District, Nanjing 210023, Jiangsu, P. R. China. Phone: +86 25 8589 1797; Email: lchen@njnu.edu.cn.)

^**^ Shile Huang, Ph.D. (Department of Biochemistry and Molecular Biology, Louisiana State University Health Sciences Center, 1501 Kings Highway, Shreveport, LA 71103, USA. Phone: +1 318 675 7759; E-mail: shile.huang@lsuhs.edu.)

**Supplementary information**

**Supplementary figures**

**FIGURE S1**

**
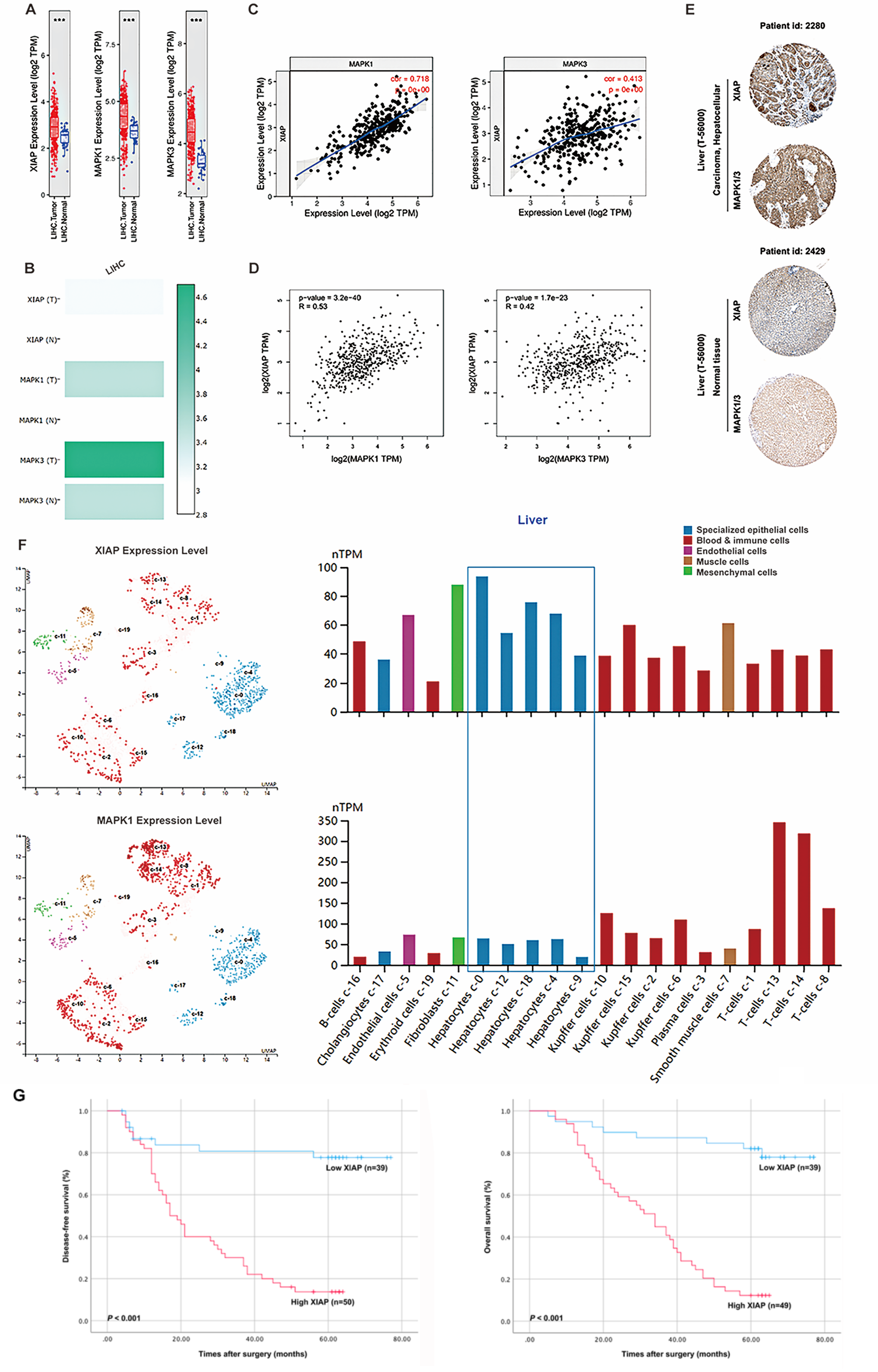
**

**FIGURE S2**

**
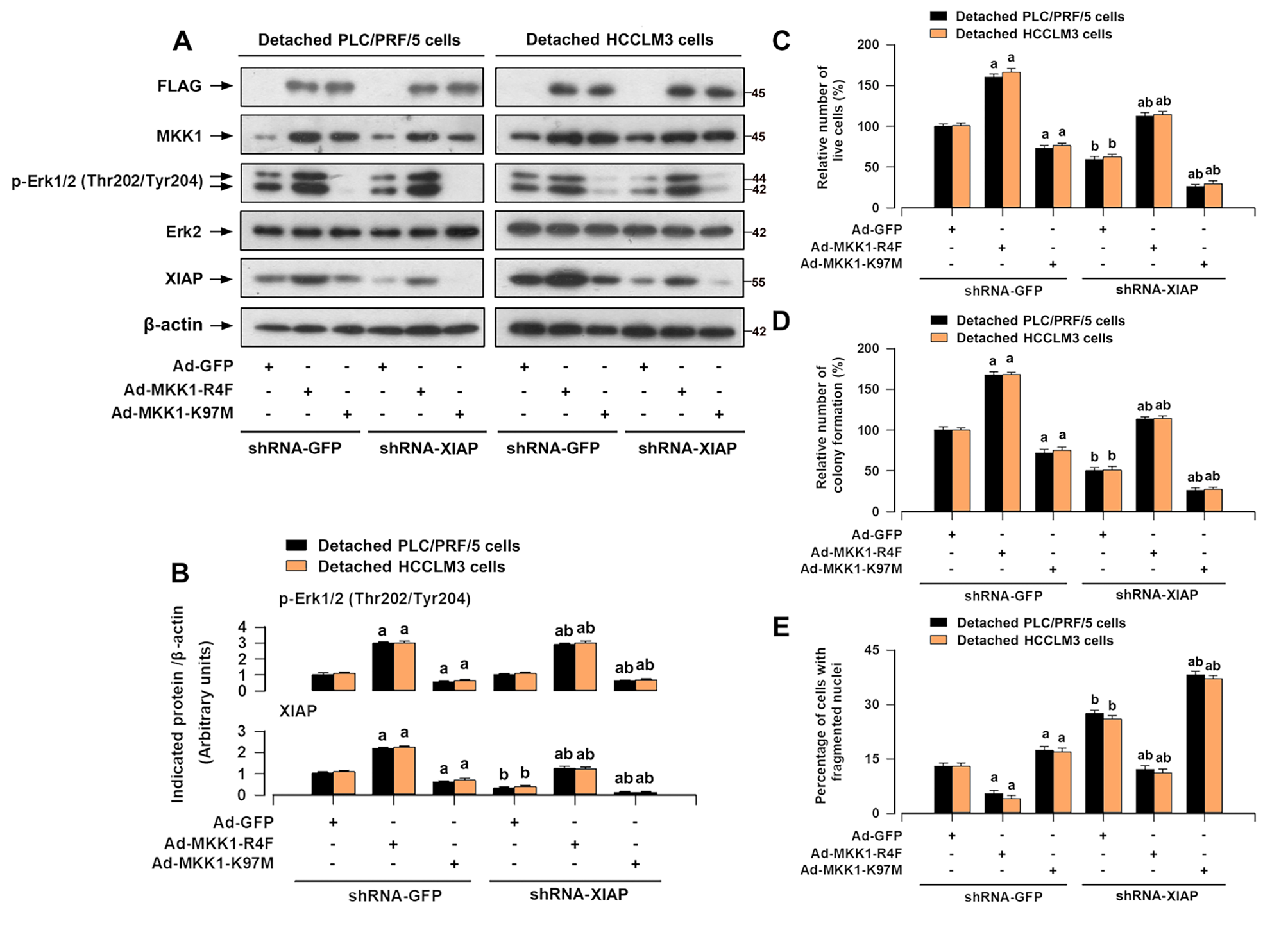
**

**FIGURE S3**

**
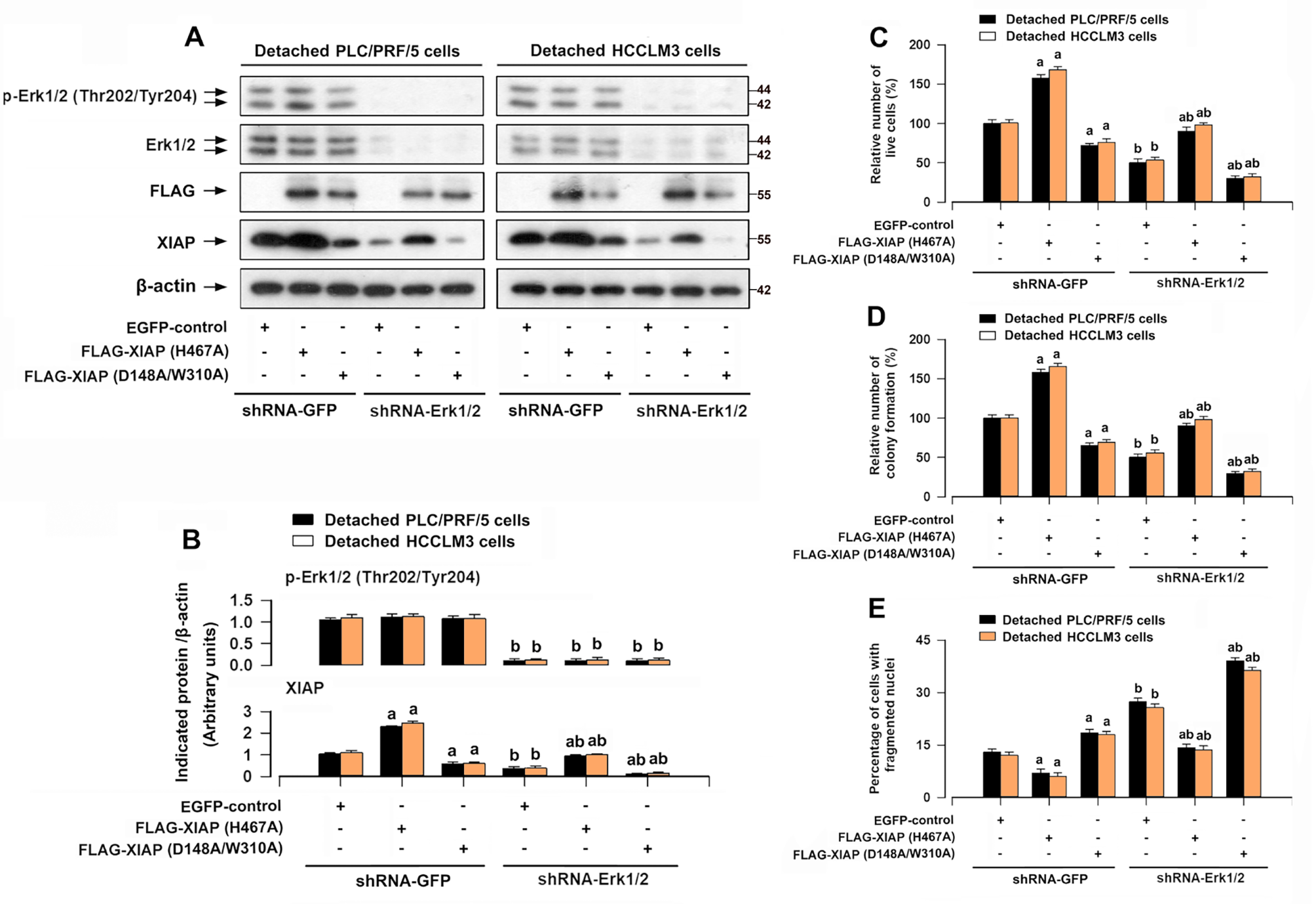
**

**Legends for supplementary figures**

**FIGURE S1** XIAP expression is positively correlated with Erk1/2 expression in HCC tissues. (A) The expression levels of XIAP (*P* < 0.001) and Erk1/2 (*P* < 0.001) between HCC and normal liver tissues from Timer database (https://cistrome.shinyapps.io/timer/) based on TCGA data. (B) The expression of XIAP (*P* < 0.001) and Erk1/2 (*P* < 0.001) in HCC tissues from GEPIA database (http://gepia.cancer-pku.cn/index.html) matched TCGA normal and GTEx data. (C) The analysis of correlation between XIAP expression and Erk1/2 expression (*r* = 0.718 or 0.413, *P* < 0.001) at mRNA level in HCC tissues using the Timer database, together with the Spearman’s rho value and estimated statistical significance. (D) The connection between XIAP expression and Erk1/2 expression (*r* = 0.53 or 0.42, *P* < 0.001) in HCC and normal liver tissues from GEPIA database, with the Spearman’s rho value and estimated statistical significance. (E) The representative IHC images of XIAP and Erk1/2 staining in HCC and normal liver tissues through Human Protein Atlas (https://www.proteinatlas.org). (F) The RNA single cell type specificity analysis of XIAP and Erk2 in different cell type proteome of the liver based on the Human Protein Atlas. (G) The correlation between the expression of XIAP and the survival rate in HCC patients was analyzed by Kaplan-Meier analysis. HCC patients with high expression of XIAP had shorter disease-free survival and overall survival.

**FIGURE S2** Ectopic expression of constitutively active or dominant negative MKK1 regulates anoikis resistance of HCC cells by XIAP. PLC/PRF/5 and HCCLM3 cells or XIAP-deficient PLC/PRF/5 and HCCLM3 cells infected with Ad-MKK1-R4F, Ad-MKK1-K97M and Ad-GFP (as control), respectively, were detached for 12 h (for Western blotting), 24 h (for trypan blue exclusion test and DAPI staining), or 2 weeks (soft agar colony formation assay). (A) The total cell lysates were subjected to Western blotting with indicated antibodies. The blots were probed for β-actin as a loading control. (B) The relative densities for p-Erk1/2 (Thr202/Tyr204) and XIAP to β-actin were semi-quantified using NIH image J. (C) The relative number of live cells was estimated by trypan blue exclusion test. (D) The cell proliferation was evaluated by soft agar colony formation assay. (E) The percentage of cells with fragmented nuclei using DAPI staining was quantified. All data were presented as mean ± SE, n = 3-5. ^a^*P* < 0.05, Ad-MKK1-R4F group or Ad-MKK1-K97M group vs Ad-GFP group; ^b^*P* < 0.05, shRNA-XIAP group vs shRNA-GFP group.

**FIGURE S3** The BIR domains of XIAP are involved in Erk1/2-dependent anoikis resistance of HCC cells. PLC/PRF/5 and HCCLM3 cells or Erk1/2-deficient PLC/PRF/5 and HCCLM3 cells infected with lentiviral FLAG-XIAP (H467A), FLAG-XIAP (D148A/W310A), or EGFP (as control), respectively, were detached for 12 h (for Western blotting), 24 h (for trypan blue exclusion test and DAPI staining), or 2 weeks (soft agar colony formation assay). (A) The total cell lysates were subjected to Western blotting with indicated antibodies. The blots were probed for β-actin as a loading control. (B) The relative densities for p-Erk1/2 (Thr202/Tyr204) and XIAP to β-actin were semi-quantified using NIH image J. (C) The relative number of live cells was estimated by trypan blue exclusion test. (D) The cell proliferation was evaluated by soft agar colony formation assay. (E) The percentage of cells with fragmented nuclei using DAPI staining was quantified. All data were presented as mean ± SE, n = 3-5. ^a^*P* < 0.05, FLAG-XIAP (H467A) group or FLAG-XIAP (D148A/W310A) group vs EGFP-control group; ^b^*P* < 0.05, shRNA-Erk1/2 group vs shRNA-GFP group.

**Supplementary tables**

Table S1 The sequences of primers for XIAP and 18s

| Name | Forward (5′-3′) | Reverse (5′-3′) |
| --- | --- | --- |
| XIAP | ACCGTGCGGTGCTTTAGTT | TGCGTGGCACTATTTTCAAGATA |
| 18s | CGCGGTTCTATTTTGTTGGT | TCGTCTTCGAAACTCCGACT |

Table S2. The sequences of primers for indicated oligonucleotides

| Name | Sequence (5′-3′) |
| --- | --- |
| XIAP sense | AATTCCCGCTTTAGGTGAAGGTGATAAATGCAAGAGATTTATCACCTTCACCTAAAGCTTTTTG |
| XIAP anti-sense | GATCCAAAAAGCTTTAGGTGAAGGTGATAAATCTCTTGCATTTTATCACCTTCACCTAAAGCGGG |
| FLAG-XIAP (S87A) Fragment 1 sense | CGGAATTCATGGATTACAAGGATGACGACGATAAGATGACTTTTAACAGTTTTGA |
| FLAG-XIAP (S87A) Fragment 1 anti-sense | GGCTATTCTCCTGTGTCTTCCAACAGCT |
| FLAG-XIAP (S87A) Fragment 2 sense | TTGGAAGACACAGGAGAATAGCCCCAAATTGCAGATTT |
| FLAG-XIAP (S87A) Fragment 2 anti-sense | CGGGATCCGTTAAGACATAAAAATTTTTTGCTTGAACGTAATGA |
| FLAG-XIAP (S87D) Fragment 1 sense | CGGAATTCATGGATTACAAGGATGACGACGATAAGATGACTTTTAACAGTTTTGA |
| FLAG-XIAP (S87D) Fragment 1 anti-sense | GTCTATTCTCCTGTGTCTTCCAACAGC |
| FLAG-XIAP (S87D) Fragment 2 sense | GACACAGGAGAATAGACCCAAATTGCAGAT |
| FLAG-XIAP (S87D) Fragment 2 anti-sense | CGGGATCCGTTAAGACATAAAAATTTTTTGCT |
| FLAG-XIAP (H467A) Fragment 1 sense | CGGAATTCATGGATTACAAGGATGACGACGATAAGATGACTTTTAACAGTTTTGA |
| FLAG-XIAP (H467A) Fragment 1 anti-sense | AGCTCCACAAGGAACAAAAACTATAGCA |
| FLAG-XIAP (H467A) Fragment 2 sense | TAGTTTTTGTTCCTTGTGGAGCTCTGGTCACTTGTAAACAGTG |
| FLAG-XIAP (H467A) Fragment 2 anti-sense | CGGGATCCGTTAAGACATAAAAATTTTTTGCTTGAACGTAATGA |
| FLAG-XIAP (D148A/W310A) Fragment 1 sense | CGGAATTCATGGATTACAAGGATGACGACGATAAGATGACTTTTAACAGTTTTGA |
| FLAG-XIAP (D148A/W310A) Fragment 1 anti-sense | AGCTACAACCTGTCCAGTTCTCAGG |
| FLAG-XIAP (D148A/W310A) Fragment 2 sense | GAGAACTGGACAGGTTGTAGCTATTTCAGATACC |
| FLAG-XIAP (D148A/W310A) Fragment 2 anti-sense | CGCATCCGTGAGCCCTCCTC |
| FLAG-XIAP (D148A/W310A) Fragment 3 sense | GAGGGCTCAC GGATGCGAAG CCAAGTGAAG |
| FLAG-XIAP (D148A/W310A) Fragment 3 anti-sense | CGGGATCCGTTAAGACATAAAAATTTTTTGCTTGAACGTAATGA |

**Supplementary methods and materials**

**Reagents**

DMEM (Cat. #11965092) and RPMI 1640 (Cat. #11875093) were supplied by Invitrogen (Grand Island, NY, USA). Fetal bovine serum (FBS, Cat. #SH30088.03) and 0.05% Trypsin-EDTA (Cat. #SH30236.01) were purchased from Hyclone (Logan, UT, USA). Embelin (Cat. #sc-201555) was obtained from Santa Cruz Biotechnology (Dallas, TX, USA). U0126 (Cat. #662005) were acquired from Sigma (St Louis, MO, USA). The antibodies utilized are as follows: p-XIAP (Ser87) (Cat. #EPR572(N), Abcam, Cambridge, UK), XIAP (Cat. #sc-55551, Santa Cruz Biotechnology), ERK2 (Cat. #sc-81457, Santa Cruz Biotechnology), β-actin (Cat. #sc-47778, Santa Cruz Biotechnology), FLAG (Cat. #B3111, Sigma), MKK1(Cat. #07-641, Sigma), ERK1/2 (Cat. #33367, Signalway Antibody, Baltimore, MD, USA), p-ERK1/2 (Thr202/Tyr204) (Cat. #9101, Cell Signaling Technology, Danvers, MA, USA), cleaved PARP (Cat. #9541, Cell Signaling Technology), and cleaved caspase-3 (Cat. #9661, Cell Signaling Technology).

**Immunohistochemistry (****IHC) staining**

Liver samples were acquired from patients who had undergone resection of HCC at Huai'an First People's Hospital. Patients received no adjuvant therapy prior to surgery. The paraffin samples were sliced into 4-μm sections and then were retrieved by Tris-EDTA buffer solution and stained overnight with primary antibodies. Photographs of IHC staining were captured under a light microscope (400×) (Leica DMi8, Wetzlar, Germany).

**Assays for cell viability and trypan blue exclusion**

The indicated cells were seeded at a concentration of 1 × 10^4^ cells/well in either 96-well normal plates or ultra-low-attachment plates and incubated for 24 h. Afterwards, the cell viability was assessed by MTS reagent following the instructions. In addition, the cells were planted at a concentration of 3 × 10^5^ cells per well in 24-well normal or ultra-low-attachment plates for 24 h. Then, the number of viable cells was quantified using the trypan blue exclusion assay.

**Western blotting**

Cells were swiftly washed with cold PBS after treatment. On ice, cells were lysed with RIPA Lysis Buffer (Cat. #PC101, Epizyme, Shanghai, China). The lysates underwent sonication for 10 s and were then centrifuged at 12000 *g* for 15 min at 4°C. Equal amounts of protein were separated by 10 % SDS-PAGE and then transferred to polyvinylidene fluoride membranes (Cat. #IPVH00010, Merck Millipore, Billerica, MA, USA). The membranes were blocked with 5% bovine serum albumin (Cat. # SW3015, Solarbio, Beijing, China) for 1 h, followed by incubation with primary antibodies at 4°C overnight and then with secondary antibodies at room temperature for 1 h. β-actin served as a loading control. Antigen-antibody complexes were detected by the ECL western blotting detection system (Merck Millipore, Burlington, MA, USA).

**Quantitative real-time PCR (qRT-PCR)**

The RNeasy RNA isolation Kit (Cat. #74104, Qiagen, Valencia, CA, USA) was utilized to extract the entire RNA, which was then reverse transcribed using the SuperScript First-strand synthesis system (Cat. #18080051, Invitrogen, Grand Island, NY, USA). The reactions were conducted with SYBR Green real-time PCR Kit (Cat. #Q221-01, Vazyme, Nanjing, China). The qRT-PCR primers listed in Supporting Information S1: Table S1 were from PrimerBank database (http://pga.mgh.harvard.edu/primerbank/). House-keeping control gene 18s was selected as an internal control. The comparative cycle threshold method (ΔΔCt method) was used to evaluate the relative expression of XIAP.

**Caspase-3/7** **activity and** **DAPI/TUNEL staining**

HCC cells were plated at a concentration of 1 × 10^4^ cells per well in 96-well normal plates or ultra-low-attachment plates for 24 h. Then, caspase-3/7 activity of the cells was quantified by Caspase-Glo®3/7 activity Kit (Cat. #G8091, Promega, Madison, WI, USA). In addition, HCC cells were cultured in 24-well normal plates or ultra-low-attachment plates at a concentration of 3 × 10^5^ cells per well. After indicated treatments, the cells were treated with DAPI. In some cases, the TUNEL staining was conducted before DAPI staining, following the instructions of TUNEL BrightRed Apoptosis Detection Kit (Cat. #A113-03, Vazyme, Nanjing, China). Fluorescence microscope (400×) (Leica DMi8, Wetzlar, Germany) was used to captured images. For quantitative analysis of TUNEL staining in HCC cells, the Image-Pro Plus 6.0 software (Media Cybernetics Inc., Newburyport, MA, USA) was utilized to determine the integral optical density.

**Supplementary images of blots**

**IMAGE S1**

**
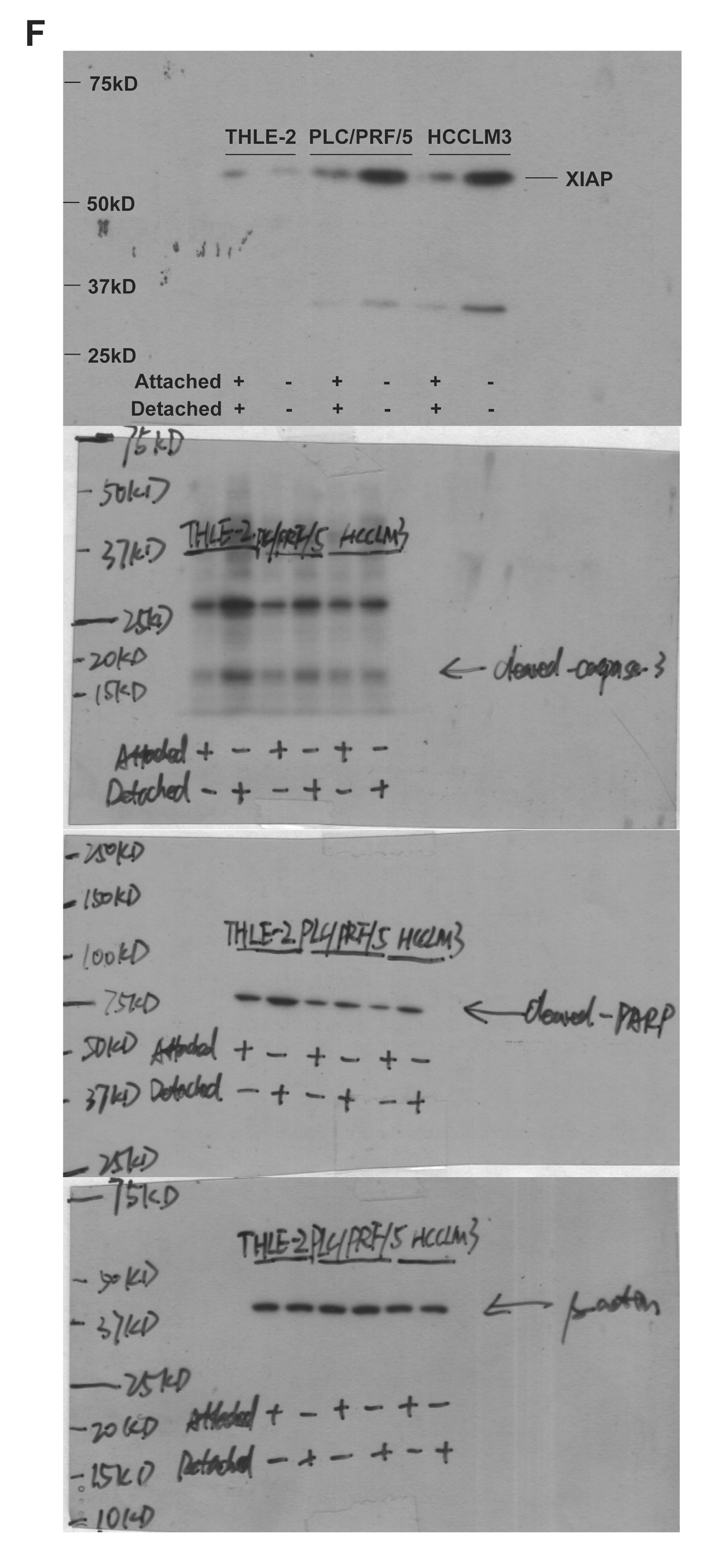
**

**IMAGE S2**

**
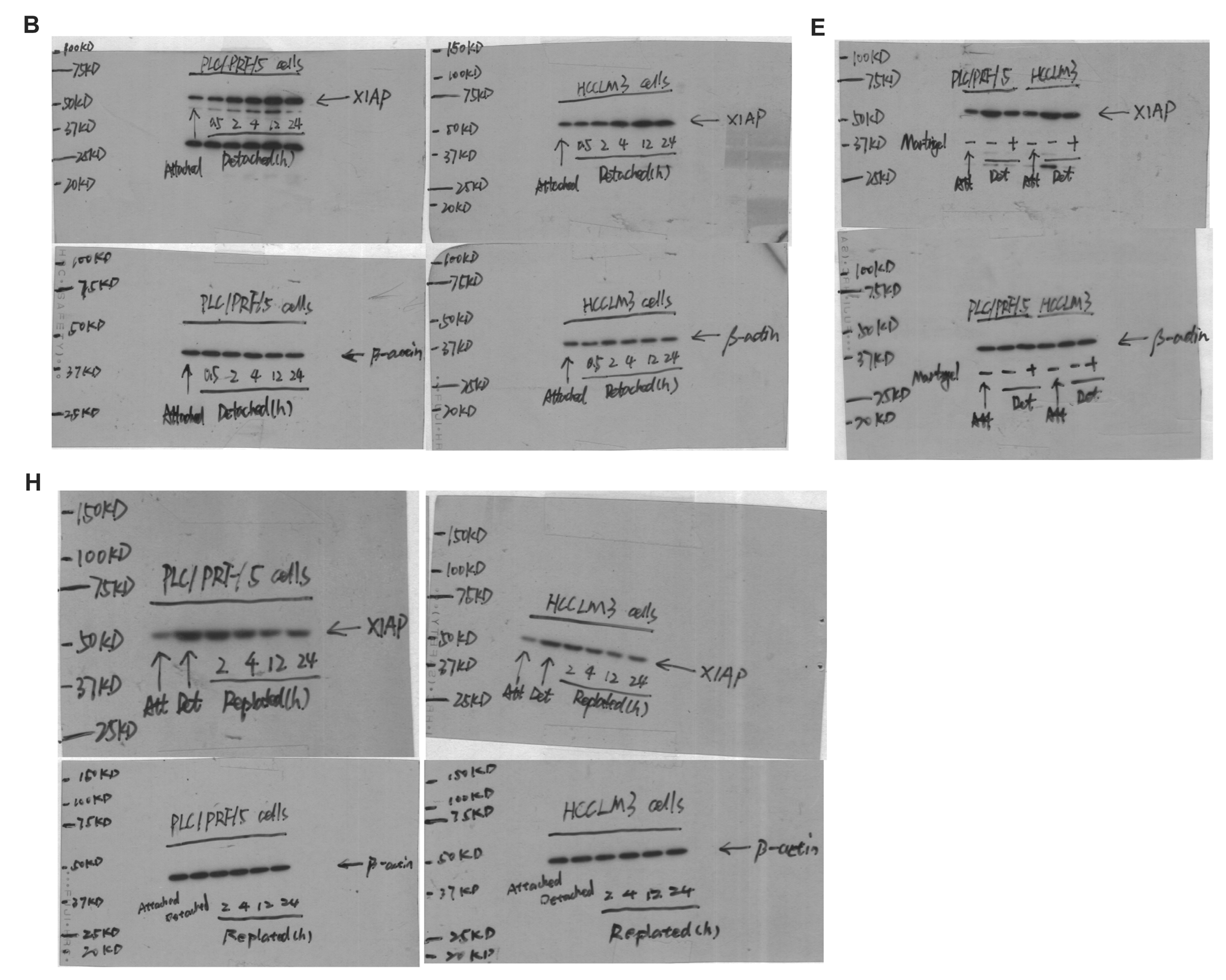
**

**IMAGE S3**

**
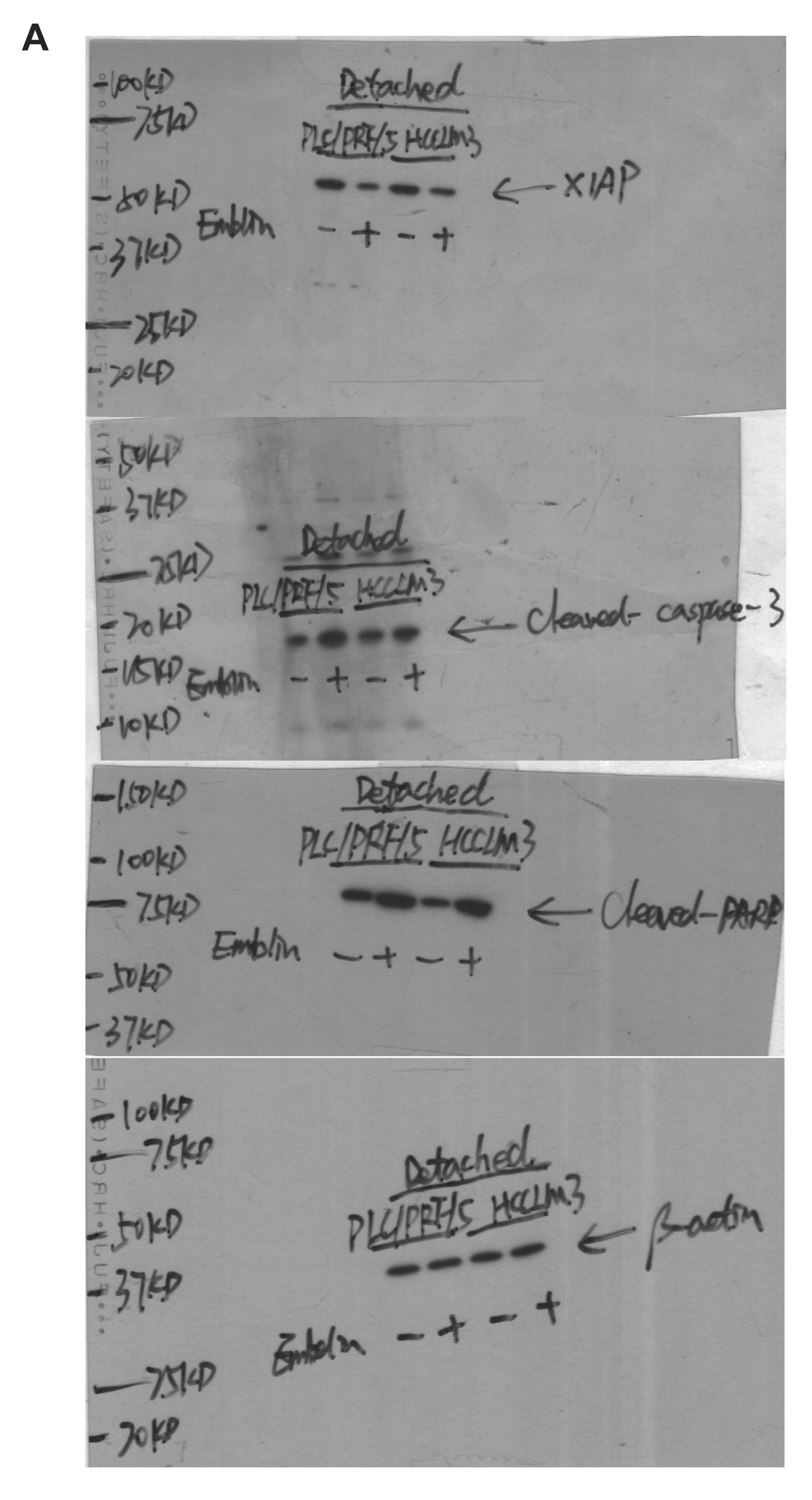
**

**IMAGE S4**

**
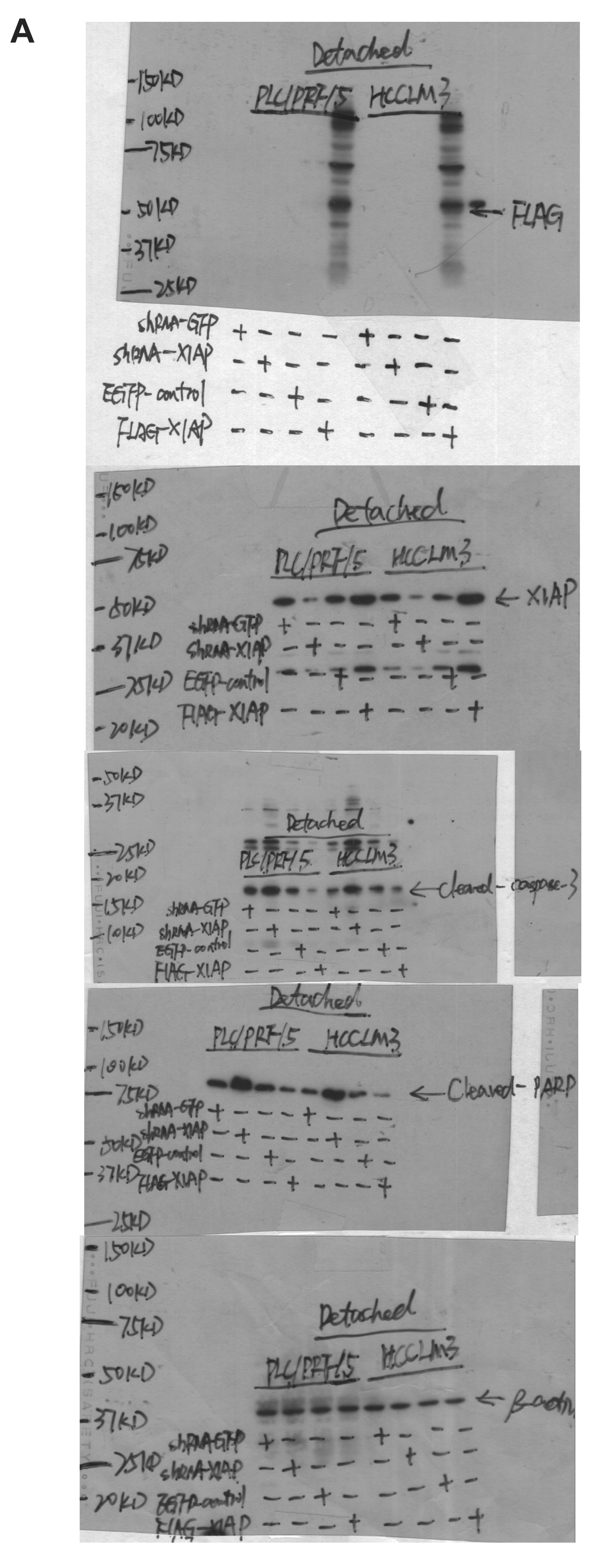
**

**IMAGE S5**

**
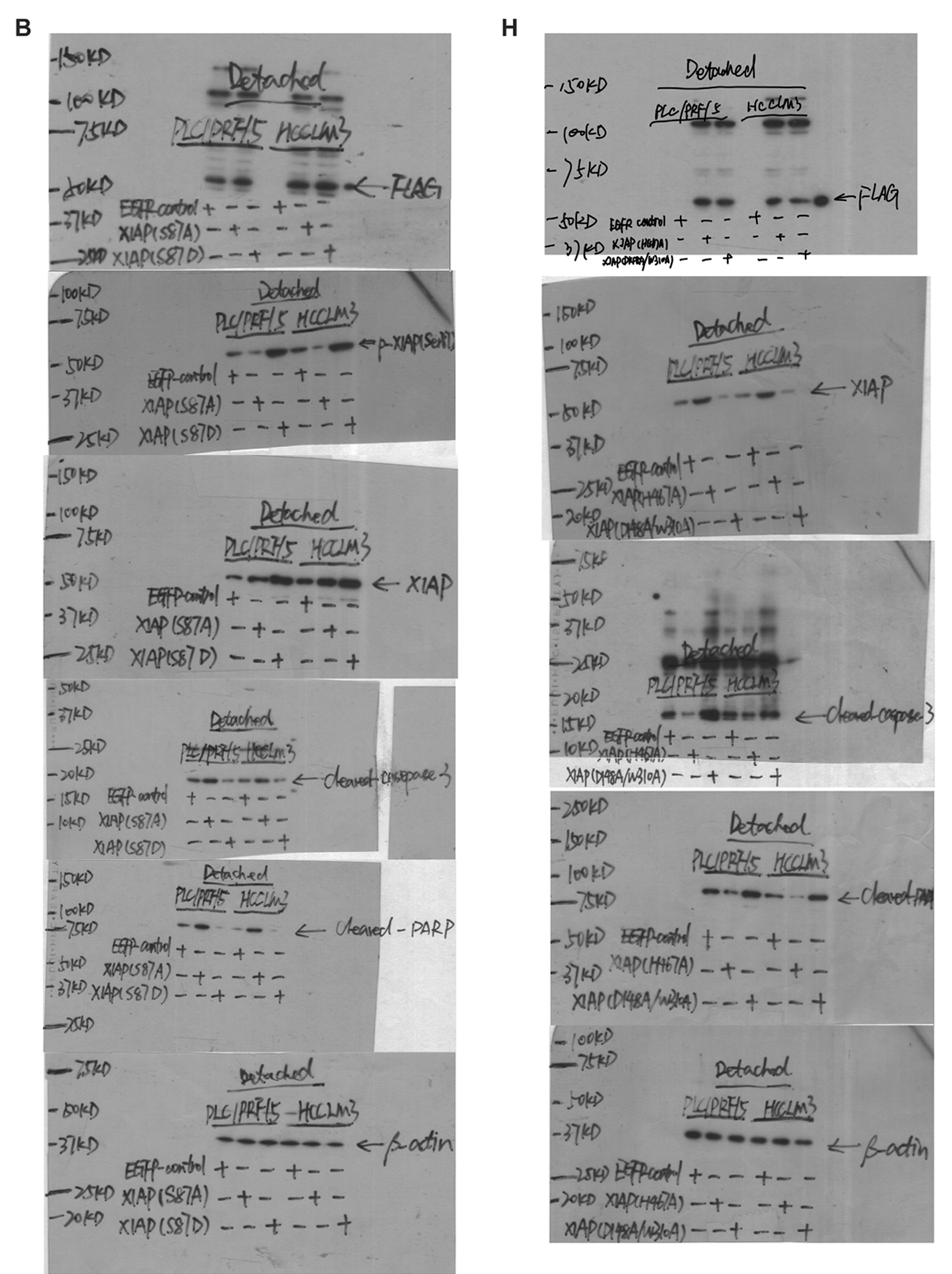
**

**IMAGE S6**

**
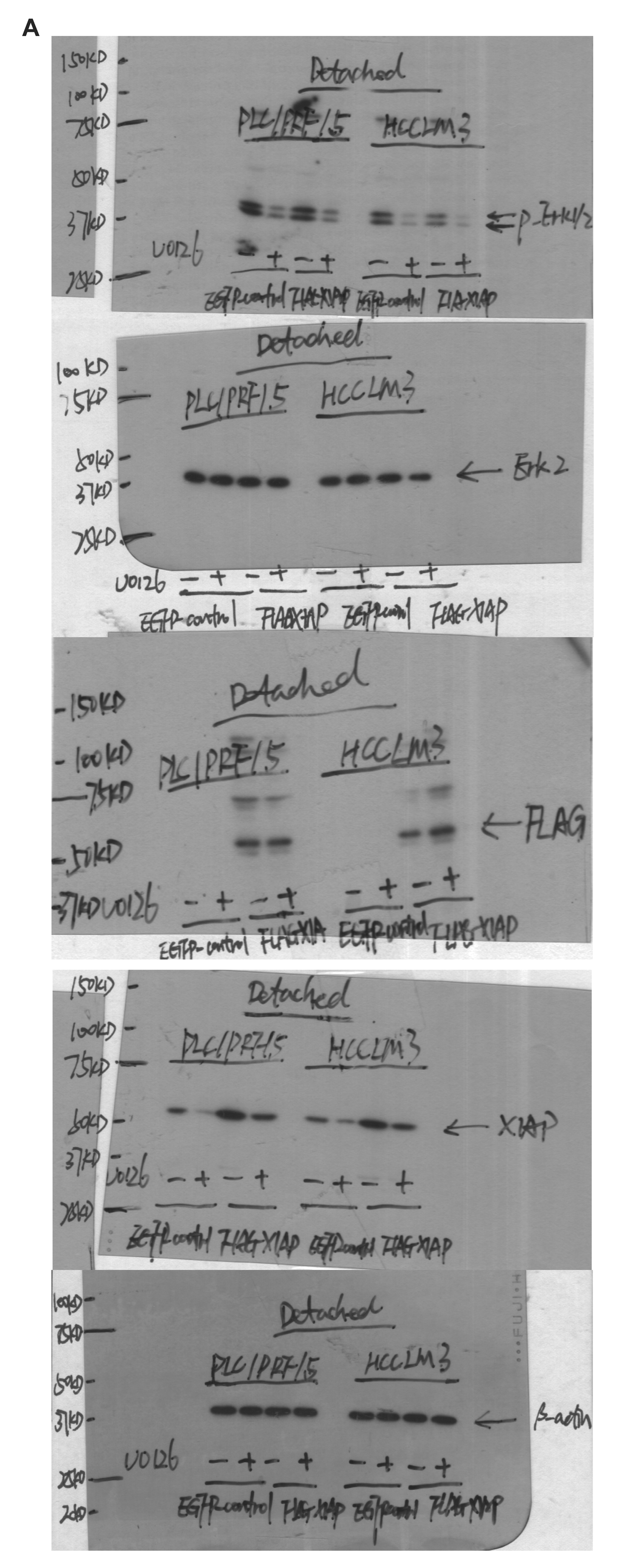
**

**IMAGE S7**

**
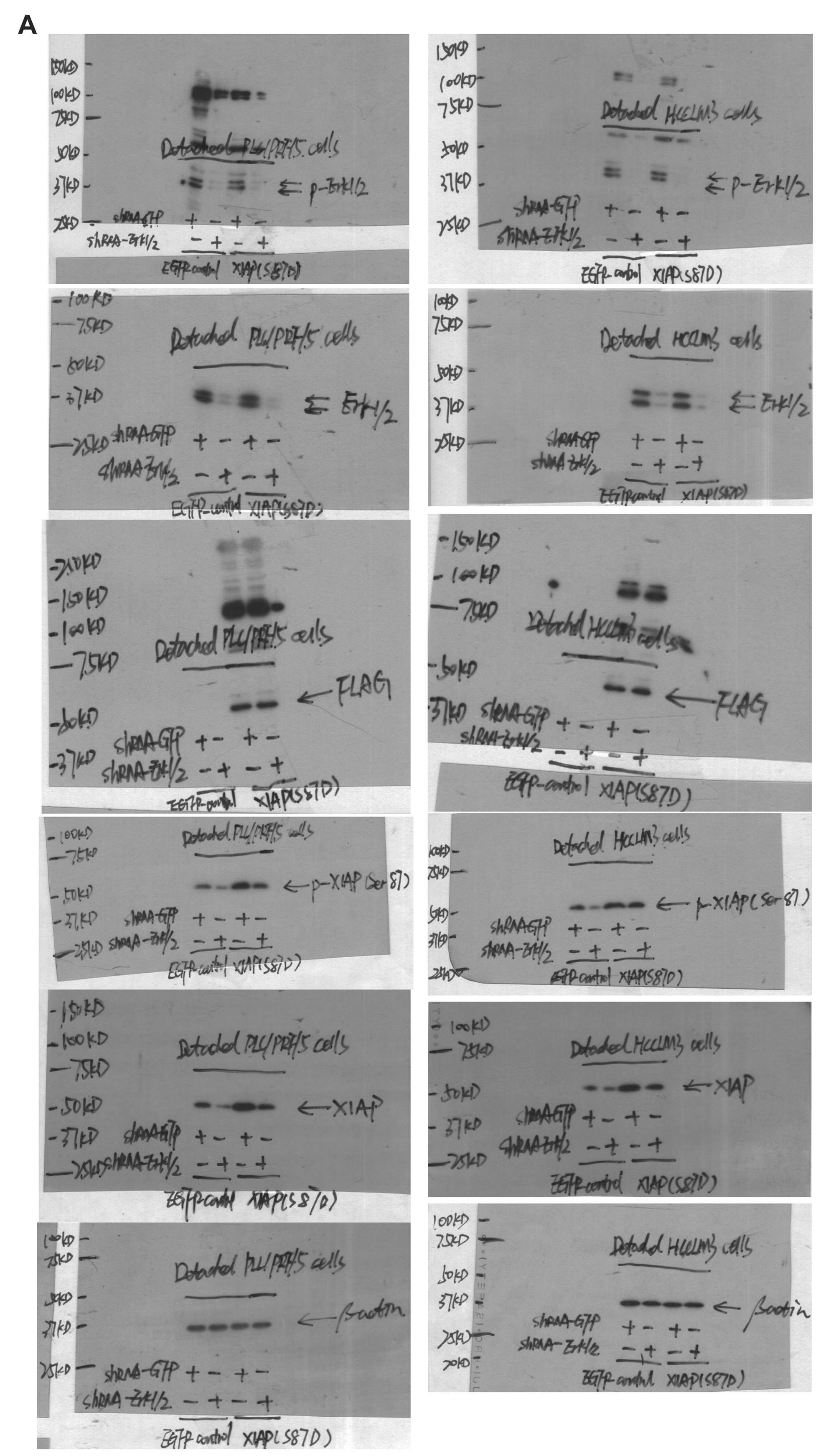
**

**IMAGE S8**

**
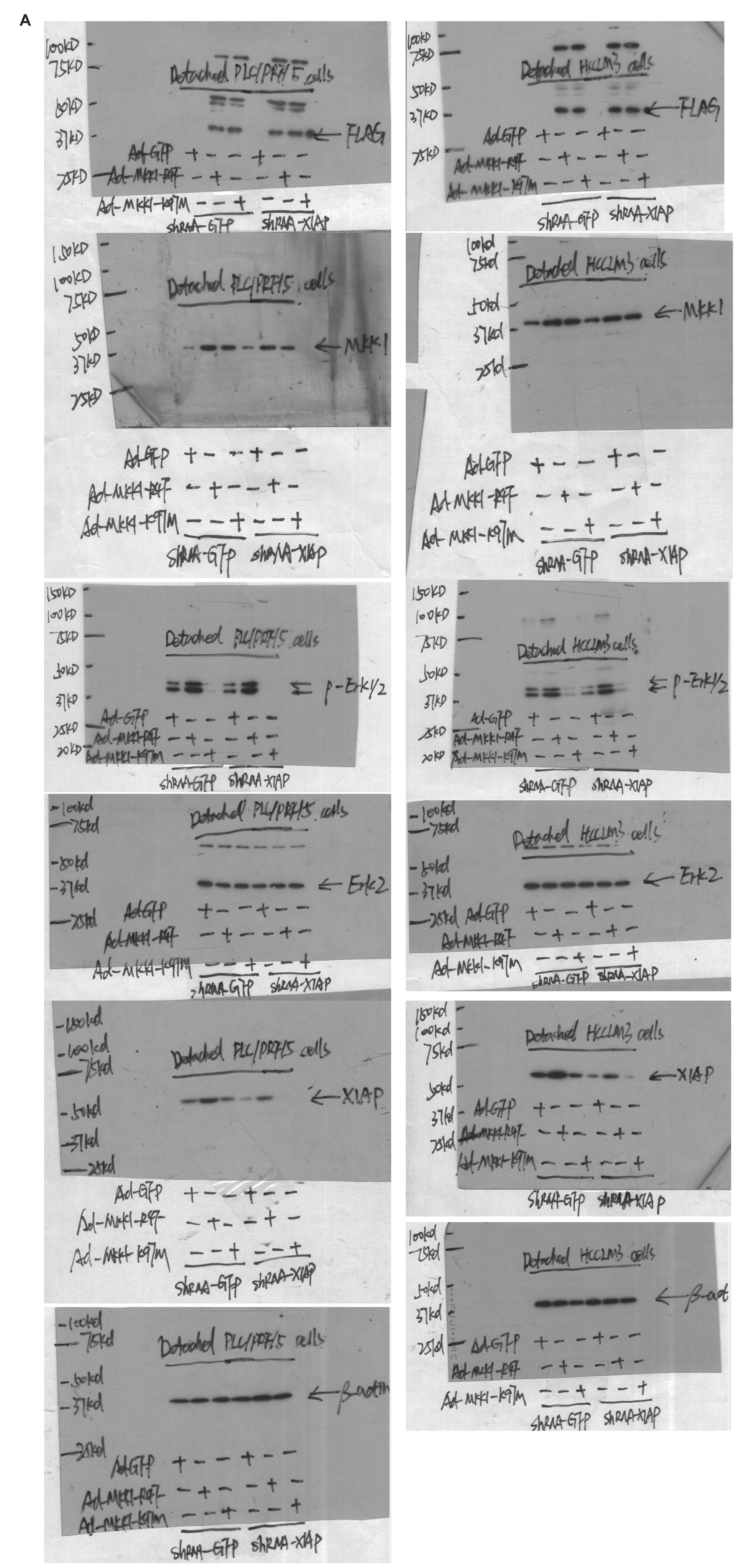
**

**IMAGE S9**

**
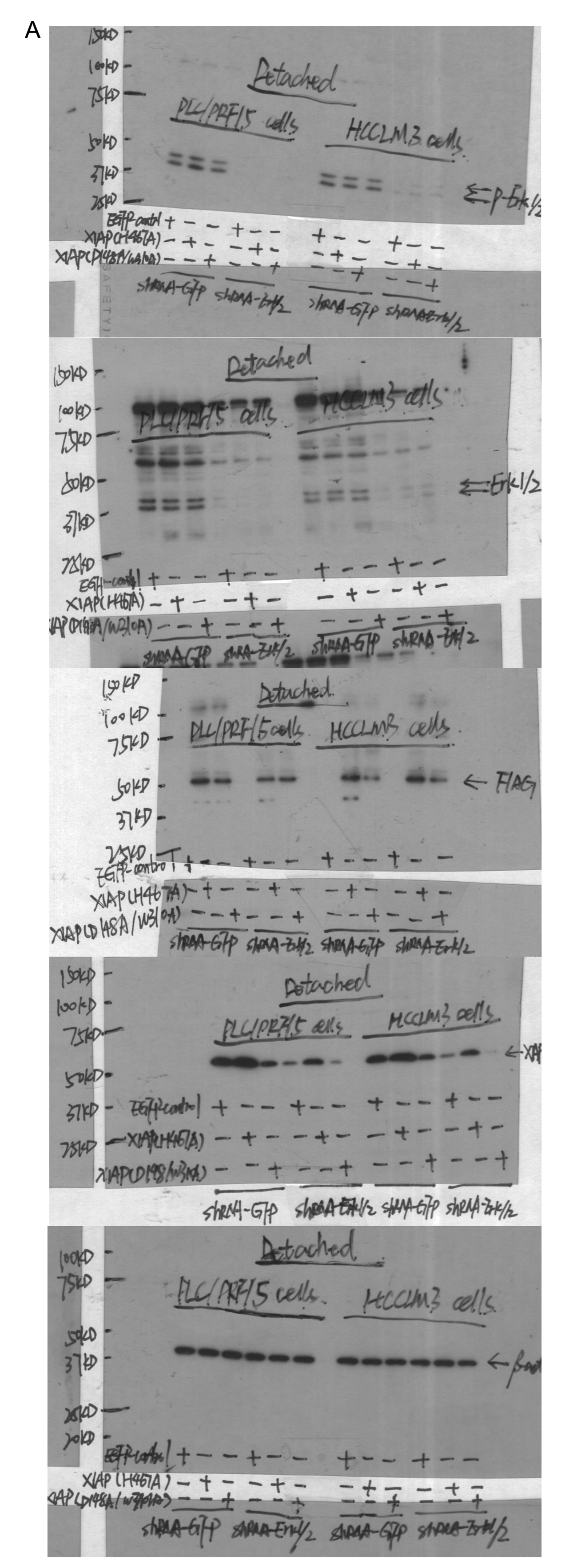
**

**Legends for supplementary images of blots**

**IMAGE S1** **Untruncated images of blots in Figure 1F**. THLE-2, PLC/PRF/5 and HCCLM3 were cultured in normal plates to allow attachment for 12 h, or transferred to ultra-low-attachment plates to induce detachment for 12 h. Total cell lysates were subjected to Western blotting using indicated antibodies. The blots were probed for β-actin as a loading control.

**IMAGE S2**  **Untruncated images of blots in Figure 2B, E and H**. PLC/PRF/5 and HCCLM3 cells were attached for 12 h, detached for the indicated time periods, pretreated with/without Matrigel followed by 12 h detachment, or detached for 12 h and then replated on normal plates for indicated time, respectively. Total cell lysates were subjected to Western blotting using indicated antibodies. The blots were probed for β-actin as a loading control.

**IMAGE S3 Untruncated images of blots in Figure 3A**. HCCLM3 and PLC/PRF/5 cells were detached for 12 h with/without 1-h pretreatment with Embelin (20 μM). Total cell lysates were subjected to Western blotting using indicated antibodies. The blots were probed forβ-actin as a loading control.

**IMAGE S4 Untruncated images of blots in Figure 4A.** PLC/PRF/5 and HCCLM3 cells infected with lentiviral shRNA to XIAP or GFP (as control), and lentiviral FLAG-tagged wild-type XIAP (FLAG-XIAP) or EGFP (as control), respectively, were detached for 12 h. Total cell lysates were subjected to Western blotting using indicated antibodies. The blots were probed for β-actin as a loading control.

**IMAGE S5 Untruncated images of blots in Figure 5B and H**. PLC/PRF/5 and HCCLM3 cells infected with lentiviral FLAG-XIAP (S87A), FLAG-XIAP (S87D), FLAG-XIAP (H467A), FLAG-XIAP (D148A/W310A), or EGFP (as control), respectively, were detached for 12 h. Total cell lysates were subjected to Western blotting using indicated antibodies. The blots were probed for β-actin as a loading control.

**IMAGE S6 Untruncated images of blots in Figure 6A**. HCCLM3 and PLC/PRF/5 cells or XIAP-overexpressing HCCLM3 and PLC/PRF/5 cells, pretreated with/without U0126 (5 μM) at indicated concentrations for 1 h, and then detached for 12 h. Total cell lysates were subjected to Western blotting using indicated antibodies. The blots were probed for β-actin as a loading control.

**IMAGE S7 Untruncated images of blots in Figure 7A**. HCCLM3 and PLC/PRF/5 cells or mutant XIAP (S87D)-overexpressing PLC/PRF/5 and HCCLM3 cells, infected with lentiviral shRNA to ERK1/2 or GFP (as control), respectively, were detached for 12 h. Total cell lysates were subjected to Western blotting using indicated antibodies. The blots were probed for β-actin as a loading control.

**IMAGE S8 Untruncated images of blots in Figure S2A**. PLC/PRF/5 and HCCLM3 cells or XIAP-deficient PLC/PRF/5 and HCCLM3 cells infected with Ad-MKK1-R4F, Ad-MKK1-K97M and Ad-GFP (as control), respectively, were detached for 12 h. Total cell lysates were subjected to Western blotting using indicated antibodies. The blots were probed for β-actin as a loading control.

**IMAGE S9 Untruncated images of blots in Figure S3A**. PLC/PRF/5 and HCCLM3 cells or Erk1/2-deficient PLC/PRF/5 and HCCLM3 cells infected with lentiviral FLAG-XIAP (H467A), FLAG-XIAP (D148A/W310A), or EGFP (as control), respectively, were detached for 12 h. Total cell lysates were subjected to Western blotting using indicated antibodies. The blots were probed for β-actin as a loading control.
